# A pancreatic cancer organoid-macrophage co-culture using starPEG-heparin hydrogel deciphers tumor-immune cell interactions

**DOI:** 10.1038/s41698-026-01678-6

**Published:** 2026-09-05

**Authors:** Julius Thyen, Victor A. Sioson, Daniel Haak, Beatrix Jahnke, Maximilian Fusenig, Abdul M. Aftab, Antonia Stammberger, Sascha Brückmann, Heike Polster, Stefanie Hübner, Verena J. Kast, Manuel Pfeifer, Rebecca Prause, Vivian Mittné, Carolin Beer, Malin H. Reinhart, Adrian M. Seifert, Lena Seifert, Martin Bornhäuser, Carsten Werner, Marc Schmitz, Daniela E. Aust, Jürgen Weitz, Daniela Loessner, Daniel E. Stange, Anna R. Poetsch, Mathieu Pecqueux, Franziska Baenke

**Affiliations:** 1 Department of Visceral, Thoracic and Vascular Surgery, Medical Faculty and University Hospital Carl Gustav Carus, TUD Dresden University of Technology, Dresden, Germany; 2 Leibniz Institute of Polymer Research Dresden, Max Bergmann Center of Biomaterials, Dresden, Germany; 3 Biomedical Genomics, Biotechnology Center, Center for Molecular and Cellular Bioengineering, TUD Dresden University of Technology, Dresden, Germany; 4 Institute of Immunology, Faculty of Medicine Carl Gustav Carus, TUD Dresden University of Technology, Dresden, Germany; 5 Institute of Pathology and Tumor- and Normal Tissue Bank of the University Cancer Center (UCC), University Hospital Carl Gustav Carus, Medical Faculty, TUD Dresden University of Technology, Dresden, Germany; 6 Institute of Legal Medicine, Medizinische Fakultät, TUD Dresden University of Technology, Dresden, Germany; 7 Core Unit for Molecular Tumor Diagnostics (CMTD), National Center for Tumor Diseases Dresden (NCT/UCC), Dresden, Germany; 8 National Center for Tumor Diseases (NCT), NCT/UCC Dresden, a partnership between DKFZ, Faculty of Medicine and University Hospital Carl Gustav Carus, TUD Dresden University of Technology and Helmholtz-Zentrum Dresden-Rossendorf (HZDR), Dresden, Germany; 9 German Cancer Consortium (DKTK), Partner Site Dresden, and German Cancer Research Center (DKFZ), Heidelberg, Germany; 10 Else Kröner Clinician Scientist Professor for Translational Tumor Immunological Research, TUD Dresden University of Technology, Dresden, Germany; 11 Medical Clinic I, University Hospital Carl Gustav Carus, TUD Dresden University of Technology, Dresden, Germany; 12 Center for Regenerative Therapies Dresden (CRTD) and Cluster of Excellence Physics of Life (PoL), TUD Dresden University of Technology, Dresden, Germany; 13 Department of Chemical and Biological Engineering and Department of Materials Science and Engineering, Faculty of Engineering, Monash University, Melbourne, VIC Australia; 14 Department of Anatomy and Developmental Biology, Biomedicine Discovery Institute, Faculty of Medicine, Nursing and Health Sciences, Monash University, Melbourne, VIC Australia; 15 Institute for Genetics, University of Cologne, Cologne, Germany; 16 Cologne Excellence Cluster for Cellular Stress Responses in Aging-Associated Diseases (CECAD), University of Cologne, Cologne, Germany; 17 Institute for Genome Stability in Aging and Disease, University of Cologne, Cologne, Germany

**Keywords:** Cancer, Immunology, Oncology

## Abstract

Macrophages are among the most abundant immune cells in the pancreatic ductal adenocarcinoma (PDAC) tumor microenvironment (TME) and play a key role in regulating the immunosuppressive niche that facilitates tumor growth. Although recent three-dimensional (3D) culture systems using patient-derived materials have advanced our understanding of tumor biology, most models lack key cellular TME components and thus fail to capture tumor-immune cell interactions. To address this gap, we developed an in-vitro 3D co-culture model incorporating PDAC patient-derived organoids (PDOs) and macrophages within a synthetic hydrogel matrix. We optimized culture conditions by tuning medium and matrix conditions to support both cell lineages. Flow cytometry and transcriptomic analyses revealed that initially undifferentiated macrophages adopt an M2-like profile upon exposure to PDAC PDOs in starPEG-heparin hydrogels, mirroring the macrophage phenotypes observed by multiplex immunohistochemistry in the matched primary PDAC tissues. Cytokine secretome profiling revealed PDO-specific differences, indicating distinct underlying macrophage polarization subtypes. Collectively, our starPEG-heparin hydrogel-based 3D co-culture enables hypothesis-driven and physiologically relevant studies of tumor-macrophage interactions and may advance immune-modulatory treatment strategies in patients with PDAC.

## Introduction

Pancreatic cancer is a highly aggressive malignancy with a dismal prognosis^1,2^. The most prevalent histological subtype is pancreatic ductal adenocarcinoma (PDAC), which accounts for more than 90% of all cases^3^. Despite advances in personalized oncology and novel therapies, surgical resection remains the only potentially curative treatment. Unfortunately, only a fraction of patients is eligible for upfront surgery at the time of diagnosis, and even among curatively resected patients, recurrence and distant metastasis occur within two years in most cases^4^. A hallmark of PDAC is its desmoplastic and immunosuppressive tumor microenvironment (TME), which is composed of an excessive extracellular matrix (ECM), cancer-associated fibroblasts (CAFs), and pro-tumorigenic immune cells, particularly tumor-associated macrophages (TAMs), which are the most abundant immune cell population^5^. The TME not only promotes tumor progression but also limits the efficacy of standard-of-care chemotherapy. In the era of immunotherapy, most preclinical studies with checkpoint inhibitors and CD40 agonists that enhance antigen presentation by macrophages and dendritic cells have shown encouraging effects in murine models. Clinical translation, however, has largely been unsuccessful^6,7^, highlighting the need for improved preclinical models that resemble the human TME. There has been enormous progress in the development of in-vitro models that mimic tumor biology. Materials obtained directly from cancer patients, hereafter referred to as patient-derived organoids (PDOs), have become valuable preclinical tools due to their ability to self-organize into 3D structures that retain genetic, molecular, and phenotypic heterogeneity of the primary tumor^8–10^. However, as PDOs are mostly of epithelial origin and lack other cell types important for the pathophysiology of the tumor, research is focusing on assembling them with additional cell types to create more physiologically relevant organoid-based models^11^. TAMs actively contribute to tumor progression by suppressing anti-tumor immunity, promoting angiogenesis, and remodeling the extracellular matrix^12^. TAMs are recruited to the TME via chemotactic signals secreted by tumor and stromal cells, and are frequently skewed toward an anti-inflammatory, M2-like phenotype. This is characterized by the expression of scavenger receptors such as CD206/MRC1 (mannose receptor C-type 1) and CD163^13,14^, although the latter can also be expressed on monocyte and tissue-resident macrophages outside of the tumor context, and is therefore not seen as an exclusive TAM marker. Macrophages are highly plastic, and their polarization is influenced by various factors, including cytokines, growth factors, hypoxia, and matrix stiffness^15,16^. TAMs suppress the anti-tumor immune response by regulating the T-cell responses and releasing immunosuppressive cytokines such as IL-10 and TGF-β^13,17^. Infiltration of TAMs is associated with poor prognosis and reduced survival in multiple tumor entities, including PDAC^18,19^. Understanding the dynamic interactions between tumor cells and TAMs is imperative to understand pathobiology and uncover mechanisms of therapeutic resistance. Progress has been made with cell culture systems that begin to capture certain aspects of this complexity, such as tumor-immune interactions. Shafran and colleagues developed an in-vitro TME model of breast cancer by co-culturing spheroids with differentiated U937-derived macrophages to evaluate drug responses^20^. Yang and colleagues reported a co-culture model of gastric cancer organoids with THP-1 macrophages to study drug-induced senescence^21^. These models are suitable for modelling tumor-immune cell interactions. Building on this, research efforts are directed towards higher physiological relevance, for instance through the incorporation of primary immune cells and alternatives to Matrigel and basement membrane extracts, as these matrices are commonly used to grow organoids but have an undefined composition and exhibit batch-to-batch variation. Therefore, synthetic hydrogel matrices with defined composition are currently evaluated for translational research^22–25^.

Here, we have established 3D co-cultures of PDAC PDOs and primary macrophages embedded in a synthetic star-shaped poly(ethyleneglycol) (starPEG)-heparin based hydrogel (starPEG-heparin hydrogel). The starPEG-heparin hydrogel, which is adapted from Kast and colleagues^26^, provides a chemically defined matrix with low batch-to-batch variation and high reproducibility. By including macrophages, it recreates the cellular components of the PDAC TME more accurately, specifically allowing the analysis of tumor-macrophage interactions. We show that PDOs and macrophages can be grown together using the same culture conditions without compromising cell viability of macrophages and the growth of PDOs. Using flow cytometry and transcriptomics, we found that naive M0 macrophages in contact with PDAC organoids undergo distinct M2-like and TAM polarization changes. Applying multiplex immunohistochemistry (mIHC) on matching primary tissues confirmed our results from our co-culture model. These findings underscore the preclinical value of our hydrogel model for deciphering the macrophage-tumor crosstalk. We identified potential macrophage-directed interventions that warrant the use of novel therapies such as cytokine inhibitors, engineered macrophages, and antibodies targeting macrophage polarization.

## Results

### Multiplex immunohistochemistry reveals an M2-like macrophage enrichment in the PDAC microenvironment

**Fig. 1:**
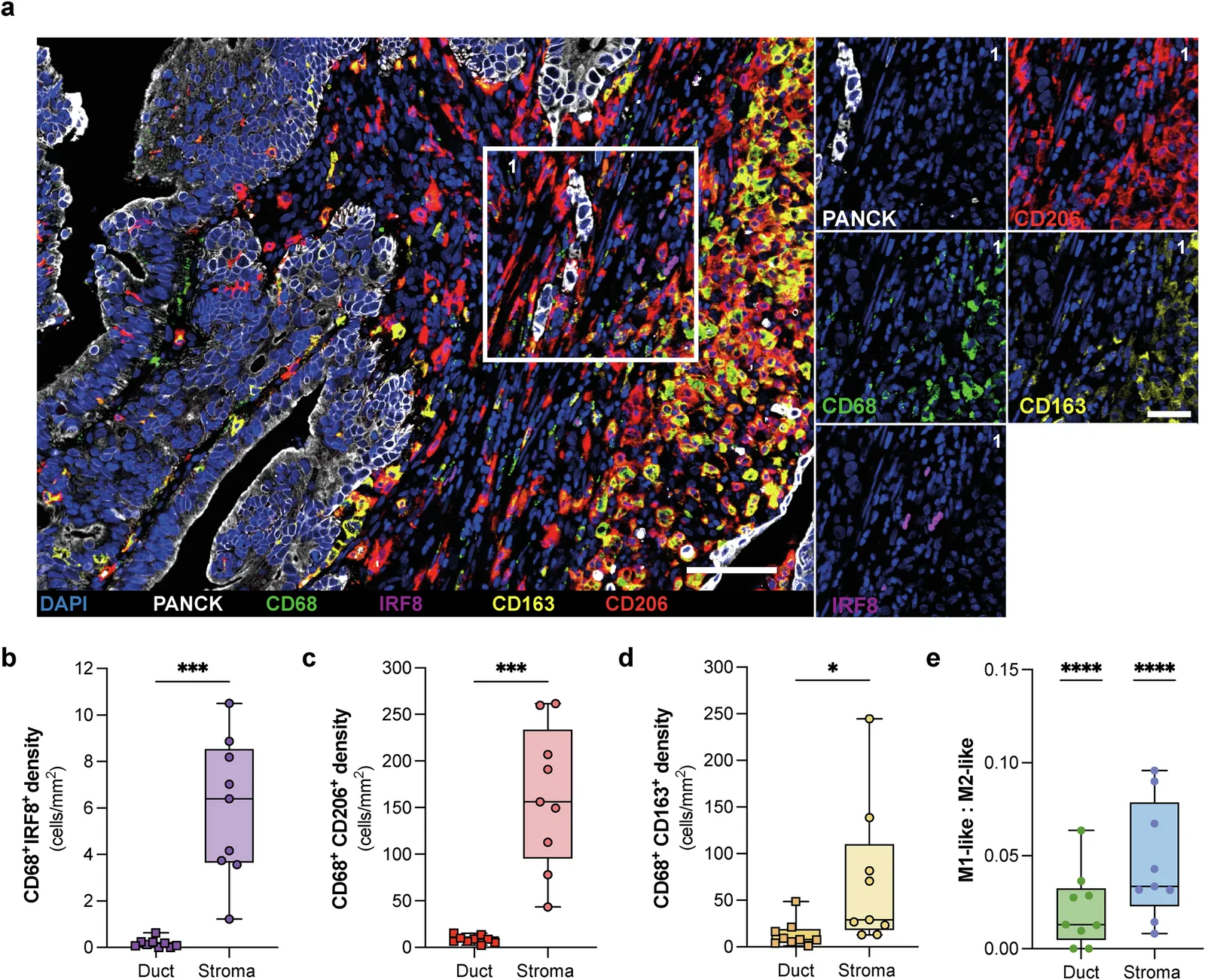
Spatial distribution of macrophages in pancreatic adenocarcinomas. **a** Representative multiplex immunohistochemistry (mIHC) image of pancreatic adenocarcinoma stained for pancreatic duct cells (PanCK in white) and conventional macrophage markers CD68 (green), IRF8 (violet), CD206 (red), CD163 (yellow), counterstained with DAPI (blue). Scale bar in overview image: 100 µm, scale bar in magnifications: 50 µm. Box-and-whisker plots depicting the median cell density (cell/mm^2^) of pan-macrophage marker CD68 co-expressing (**b**) M1-like macrophage marker IRF8 and M2-like macrophage markers (**c**) CD206 and (**d**) CD163 across ductal and stromal regions (*n* = 9). Statistical differences were identified using a paired t-test; *p < 0.05, **p < 0.01. **e** Box-Whisker plot depicting the ratio of M1-like (CD68^+^ IRF8^+^) to M2-like (CD68^+^ CD206^+^) across ductal and stromal regions. Statistical differences were identified using a one-sample *t*-test; ****p < 0.0001; *n* = 9.

Intratumoral macrophages are pivotal components of the TME in solid cancers, and they are known to impact cancer progression and influence therapy resistance^17^. Multiplex IHC staining was performed on FFPE tissue sections from nine treatment-naïve PDAC patients to assess the density and spatial distribution of intratumoral macrophages. The mIHC panel comprised four known macrophage markers - CD68 (pan-macrophage), IRF8 (M1-like), CD206 (M2-like) and CD163 (M2-like) - and pan-cytokeratin (PanCK) to distinguish tumor cells from the stroma together with DAPI for nuclear counterstaining (Fig. 1a). Quantitative image analysis revealed that the most abundant intratumoral macrophages consisted of CD68^+^ cells expressing the M2-like receptors CD206^+^ (123.38 ± 19.51 cells/mm^2^) or CD163^+^ (56.17 ± 20.32 cells/mm^2^), whereas CD68^+^ cells co-expressing the M1-like transcription factor IRF8^+^ were rare (4.56 ± 0.88 cells/mm^2^; Supplementary Figs. S1a). All assessed macrophage populations were significantly enriched in the stromal compartment compared to the ductal regions (CD68^+^: 359.12 ± 55.97 cells/mm^2^ vs. 66.18 ± 13.147 cells/mm^2^: CD68^+^ IRF8^+^: 0.18 ± 0.065 cells/mm^2^ vs. 5.96 ± 0.99 cells/mm^2^; CD68⁺ CD206⁺: 8.61 ± 1.35 cells/mm^2^ vs 162.18 ± 25.29 cells/mm^2^; and CD68⁺ CD163⁺: 13.34 ± 4.86 cells/mm^2^ vs 71.078 ± 25.70 cells/mm^2^; Fig. 1b–d). The ratio of M1-like to M2-like macrophage showed a predominance of M2-like macrophages (CD68^+^ CD206^+^) in both tissue compartments (Fig. 1e). Evaluation of the proportion of these macrophage subsets also showed CD68^+^ CD206^+^ as the most dominant population (42.74%), followed by CD68^+^ CD163^+^ macrophages (17.05%), and CD68^+^ IRF8^+^ macrophages being a rare population (1.73%) (Supplementary Figs. S1b). This pattern was maintained when comparing the proportion in the ductal or stromal compartment. The M1- and M2-like subsets were predominantly localized in the stroma region, with higher expression of M2-like macrophages, whilst the proportion of CD68^+^ macrophages co-expressing CD163 was not different between the two compartments (Supplementary Figs. S1c–e). Collectively, our results indicate that M2-like macrophages are the predominant intratumoral macrophage population in PDAC, with significantly higher density and frequency in the stroma and a uniformly low density and frequency of M1-like macrophages across compartments.

### Establishment of PDAC PDOs for 3D hydrogel co-cultures with macrophages

**Fig. 2:**
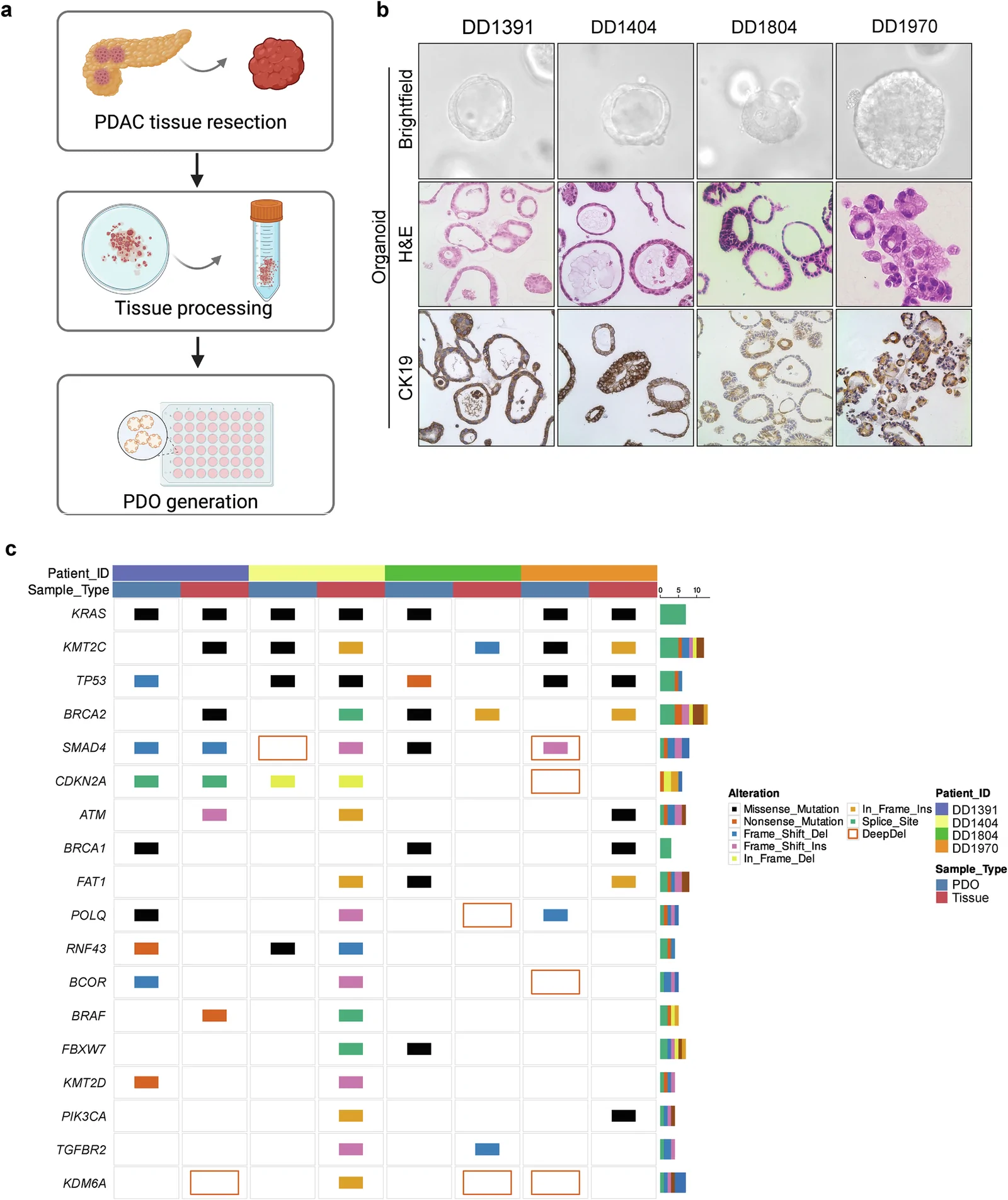
Molecular characterization of patient-derived PDAC organoids. **a** Schematic overview of the generation of PDOs from surgically resected PDAC tissue. **b** Brightfield images and immunohistochemical staining (H&E, CK19) of PDAC PDOs. Scale bar: 100 µm. **c** Oncoplot of the most frequently mutated genes in PDAC PDOs and their matching primary tissue.

To better understand the interactions of tumor cells with macrophages in PDAC, we established an in-vitro 3D co-culture model incorporating both cell types. Nine PDOs were generated from resected PDAC tissues (Fig. 2a) that matched the tissue sections analyzed by mIHC. For four PDO lines, we were able to establish long-term cultures in Matrigel and PDAC organoid medium according to standard protocols^8,10^ and used them for further downstream analysis. Each organoid exhibited a distinct morphology. DD1391 displayed predominantly cystic structures compared to the compact, grape-like structure of DD1970. DD1404 and DD1804 displayed a mixed morphology (Fig. 2b). Confirmation of the epithelial origin was carried out by the expression of epithelial marker CK19 being present in all four PDO lines and their corresponding PDAC tissue (Supplementary Fig. S2a). Next, we evaluated the mutational profile of these PDOs by applying targeted sequencing and found that all organoid lines carried mutations in *TP53* and *KRAS*, which are known key drivers in the pathogenesis of PDAC^27^. Individual organoid lines harbored other PDAC-related genes, such as *SMAD4, ERBB2*, and *CDKN2A* (Fig. 2c). The observed heterogeneity in morphology and genetic alterations across the PDAC PDOs points to their complexity and provides a rationale for modelling tumor-immune cell interactions.

### Optimized medium conditions enable the growth of PDAC PDOs and macrophages

**Fig. 3:**
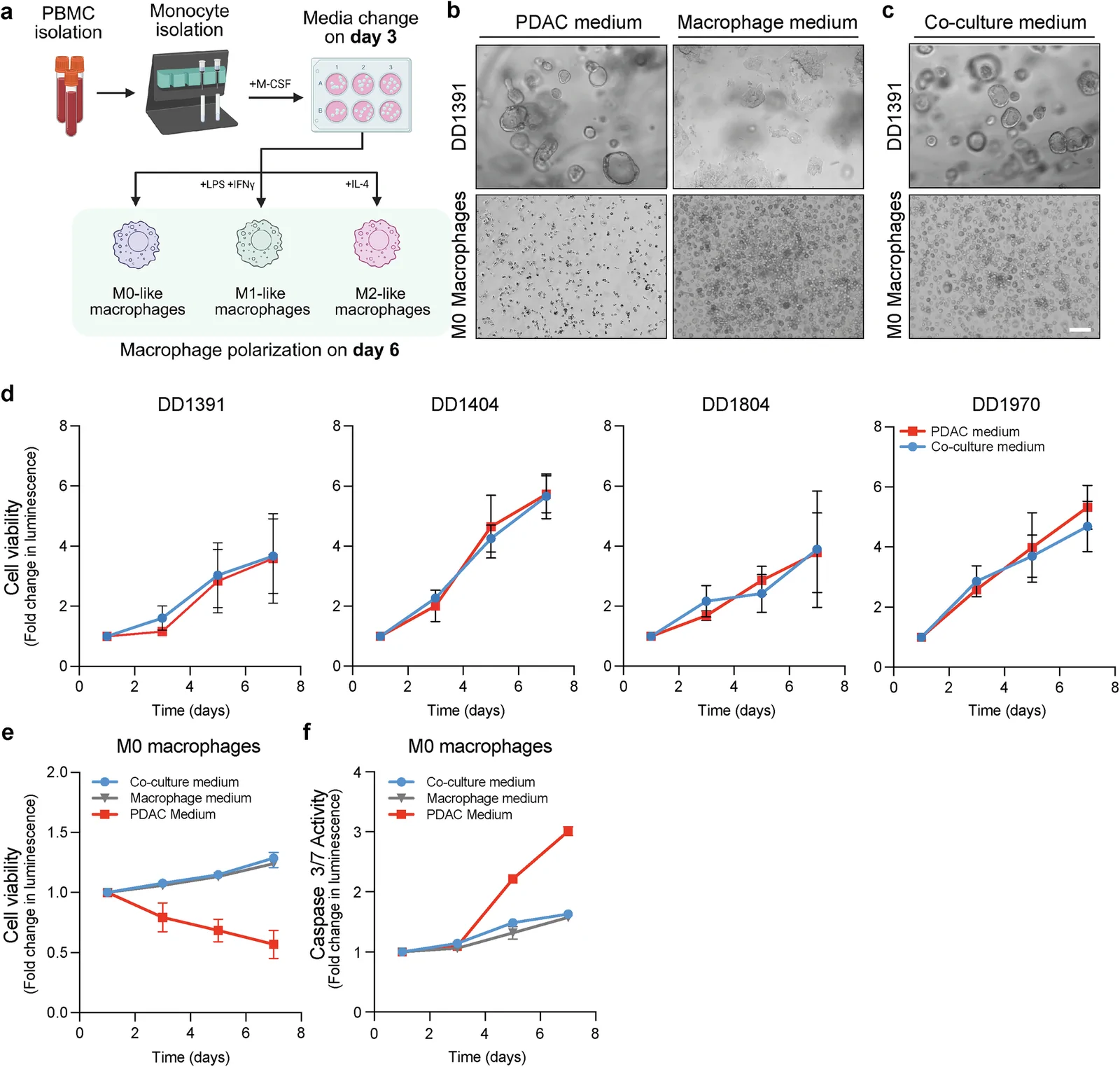
Optimization of culture media for PDAC PDOs and macrophages. **a** Schematic overview of the isolation of PBMCs from whole blood samples for the isolation of monocytes and their differentiation to macrophages using M-CSF. Polarization of macrophages was induced chemically by exposing M0 macrophages to either LPS, IFNγ for M1-like macrophages or IL-4 for M2-like macrophages. **b** Representative brightfield images of Matrigel-embedded PDAC organoid line DD1391 and M0 macrophages cultured in either standard PDAC organoid medium or macrophage medium. Scale bar: 130 µm **c** Representative brightfield images of PDAC PDO line DD1391 embedded in Matrigel and M0 macrophages cultured in co-culture medium. Scale bar: 130 µm. **d** Viability measurement of established PDAC PDO lines embedded in Matrigel, cultured in their niche PDAC organoid medium (shown in red) or co-culture medium (shown in blue). Mean values from independent biological replicates (*n* = 2, each with 3 technical replicates) per PDO line are shown, with error bars representing the standard error of the mean (SEM). **e** Viability measurement of M0 macrophages in suspension and cultured in their standard macrophage medium (shown in grey), PDAC organoid medium (shown in red), or co-culture medium (shown in blue) for 7 days. The mean values and SEM of *n* = 3 independent experiments are displayed. **f** Assessment of Caspase 3/7 activity using Caspase Glo of M0 macrophages in suspension, cultured in either their standard macrophage medium (shown in grey), co-culture medium (shown in blue) or PDAC medium (shown in red). The mean values and SEM of *n* = 3 independent experiments are displayed.

Establishing a 3D co-culture system of multiple cell types requires careful optimization of culture conditions that support the viability and growth of both cell types^28^. To explore a suitable medium for co-culturing both organoids and macrophages, which were differentiated from blood-derived monocytes of PDAC patients (Fig. 3a), we first assessed each cell type separately in the standard PDAC organoid and macrophage medium (Supplementary Table S3). As expected, PDAC PDOs embedded in Matrigel with its standard culture medium formed well-organized 3D structures. However, when cultured in macrophage medium, PDOs lost their architecture and adopted a flattened 2D morphology (Fig. 3b), indicating that macrophage medium is not suitable for sustaining organoid growth. Conversely, brightfield images revealed fewer macrophages in PDAC medium compared to macrophage medium (Fig. 3b). This reciprocal medium incompatibility highlights the distinct dependencies of tumor and immune cells. We therefore designed a co-culture medium (CCM, Supplementary Table S3) that combined essential components from both medium formulations. In this medium, PDOs retained their morphology over multiple passages with no significant differences in morphology (Fig. 3b, c, Supplementary Figs. S3a) and organoid viability (Fig. 3d) between PDAC and CCM. Macrophage viability, measured by ATP content, remained comparable between macrophage medium and CCM (Fig. 3e). By contrast, macrophages cultured in PDAC medium displayed reduced ATP levels and increased caspase 3/7 activity, consistent with stress-induced apoptosis (Fig. 3f, Supplementary Fig. S3b). Together, these data suggest that CCM enables macrophage and PDAC PDO co-existence and thereby provides a physiological environment to study tumor-macrophage interactions.

### StarPEG-heparin hydrogels promote 3D co-cultures and growth of PDOs and macrophages

**Fig. 4:**
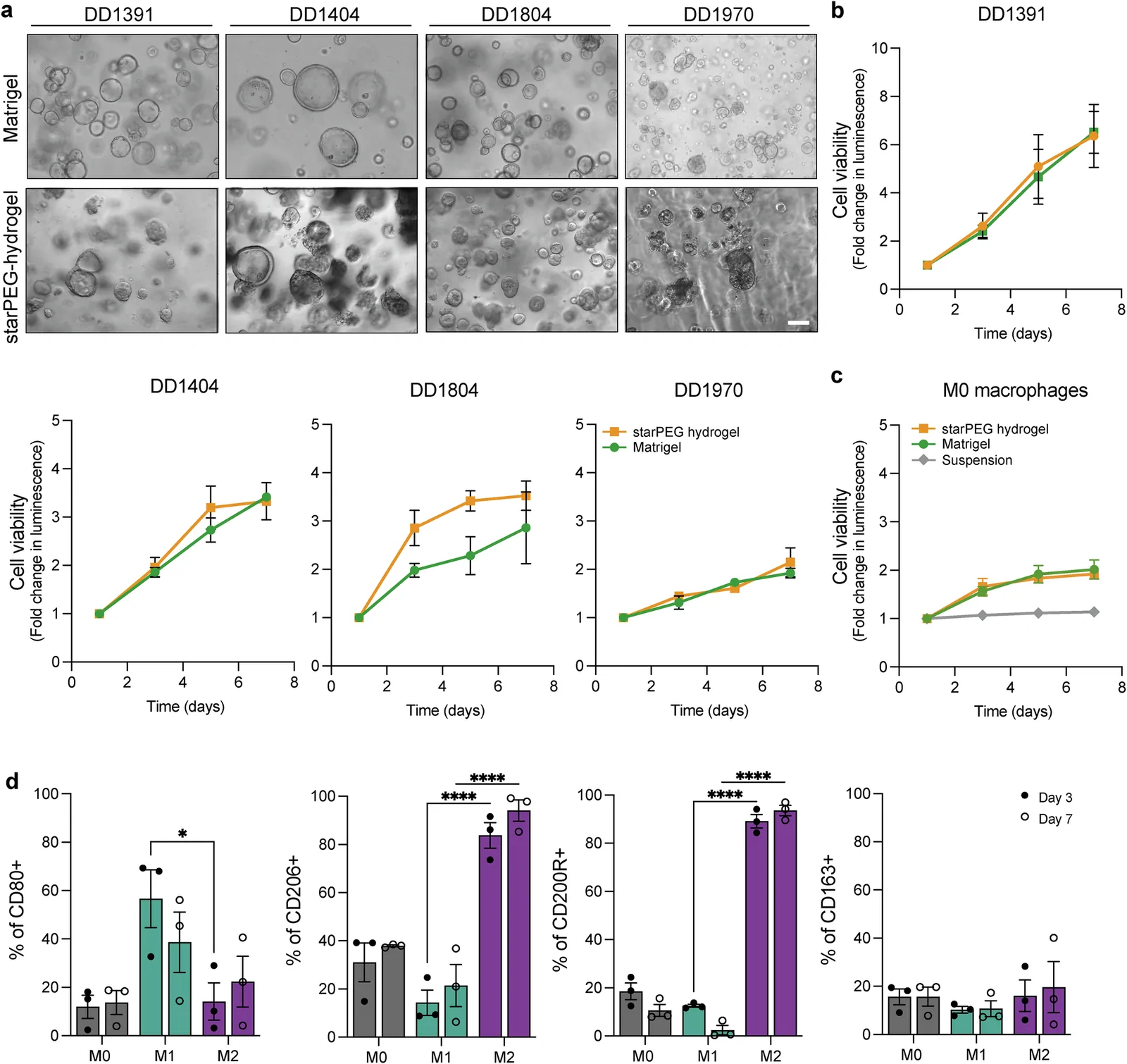
Matrix optimization for 3D co-culture conditions of macrophages and PDAC PDOs. **a** Representative brightfield images of PDAC PDO lines embedded in Matrigel or in starPEG-heparin hydrogel cultured in co-culture medium. Pictures were taken after 7 days of culture. Scale bar: 130 µm. **b** Viability measurement of four PDO lines embedded in Matrigel (shown in green) or starPEG-heparin hydrogel (shown in orange), cultured in co-culture medium for 7 days. The relative luminescence was normalized to day 1. The mean values and SEM of *n* = 3 independent replicates are displayed. **c** Viability measurements of M0 macrophages in suspension (shown in grey), embedded in Matrigel (shown in green) or starPEG-heparin hydrogel (shown in orange), cultured in co-culture medium for 7 days. The relative luminescence was normalized to day 1. The mean values and SEM of *n* = 3 independent replicates are displayed. **d** Cell surface marker expression of in-vitro differentiated M0, M1-like, and M2-like macrophages embedded in starPEG-heparin hydrogel and cultured in co-culture medium. The mean values and SEM of *n* = 3 independent replicates are displayed on day 3 (black dots) and day 7 (white dots). ANOVA, *p < 0.05, ****p < 0.0001.

Another essential component in a co-culture system is the extracellular matrix (ECM), which provides biochemical and structural cues to support the viability, adhesion, and function of the different cell types^29^. Most organoid cultures rely on matrices derived from murine tumors with unknown composition, such as Matrigel. This might limit the feasibility of these 3D organoid cultures for immunological readouts due to potential immunogenicity. To address this, we evaluated culturing PDOs and macrophages in a controlled, fully defined, and tunable starPEG-heparin hydrogel that has been reported by Kast and colleagues^26^. PDOs cultured in CCM were embedded in either Matrigel or starPEG-heparin hydrogels to assess their cell behavior. In both conditions, PDOs maintained their 3D architecture and morphology (Fig. 4a). Viability analyses showed no growth differences in PDOs cultured in either matrix for 7 days (Fig. 4b). To investigate the matrix-induced effect of starPEG-heparin hydrogel on PDAC PDO drug sensitivity, we exposed the PDAC PDOs to single chemotherapeutics being part of the current standard-of-care (e.g. 5-FU, oxaliplatin, irinotecan and gemcitabine, Supplementary Fig. S4) for six days. The drug response curves revealed no significant differences of PDO lines DD1404 and DD1970 cultured in starPEG-heparin hydrogel compared to Matrigel, except for DD1970 in response to oxaliplatin (Supplementary Fig. S4). Similarly, macrophages embedded in starPEG-heparin hydrogels did not exhibit any morphological changes compared to the macrophages in suspension, independent of polarization state (Supplementary Fig. S5a). Minimal differences in macrophage viability were seen when comparing growth in suspension compared to 3D matrices (Fig. 4c). To ensure that the function of macrophages is not compromised by using CCM and starPEG-heparin hydrogels, we examined the expression of known macrophage markers CD68, CD80, CD206, CD200R, and CD163 using flow cytometry on day 3 and day 7 (Fig. 4d, Supplementary Fig. S5b). Polarization of M0 macrophages by exposure to IFNγ and LPS to M1-like macrophages resulted in significantly elevated expression of CD80 compared to M0- and M2-like macrophages (via exposure of M0 macrophages to IL-4). This difference was not observed on day 7. Conversely, significantly more M2-like macrophages (CD206/MRC1 and CD200R) were detected. As expected, due to the absence of tumor-derived factors or tumor cell-conditioned medium in the macrophage monoculture setting, CD163 showed no significant differences between M0 and M2-like macrophages^30,31^. To determine whether the stiffness of the hydrogel matrix (i.e., the crosslinking density/ γ of starPEG to heparin) influences macrophage polarization in the co-culture setting, three stiffness levels were assessed by adjusting the starPEG-to-heparin ratio, generating softer (γ = 0.75), intermediate (γ = 1), and stiffer (γ = 1.25) hydrogels. Macrophage polarization, assessed by CD80 and CD206 expression, was similar across hydrogel stiffness conditions (γ = 0.75, 1.0, 1.25) at both day 3 and day 7 of co-culturing, with PDO-associated upregulation of CD206 and low CD80 expression across all crosslinking densities and organoid lines (Supplementary Fig. S5c). These data suggest that PDO-derived cues rather than the stiffness of the hydrogel are the dominant drivers of macrophage polarization under the investigated conditions here. Together, these results show that macrophage subtypes can be induced in a 3D hydrogel culture setup. Importantly, both cell types cultured in CCM and starPEG-heparin hydrogel retain their viability and phenotype, thus enabling the establishment of a physiologically relevant and fully defined 3D co-culture model suitable for studying tumor-immune interactions.

### 3D co–culture of PDAC PDOs and macrophages in hydrogel reveals tumor-driven macrophage polarization

**Fig. 5:**
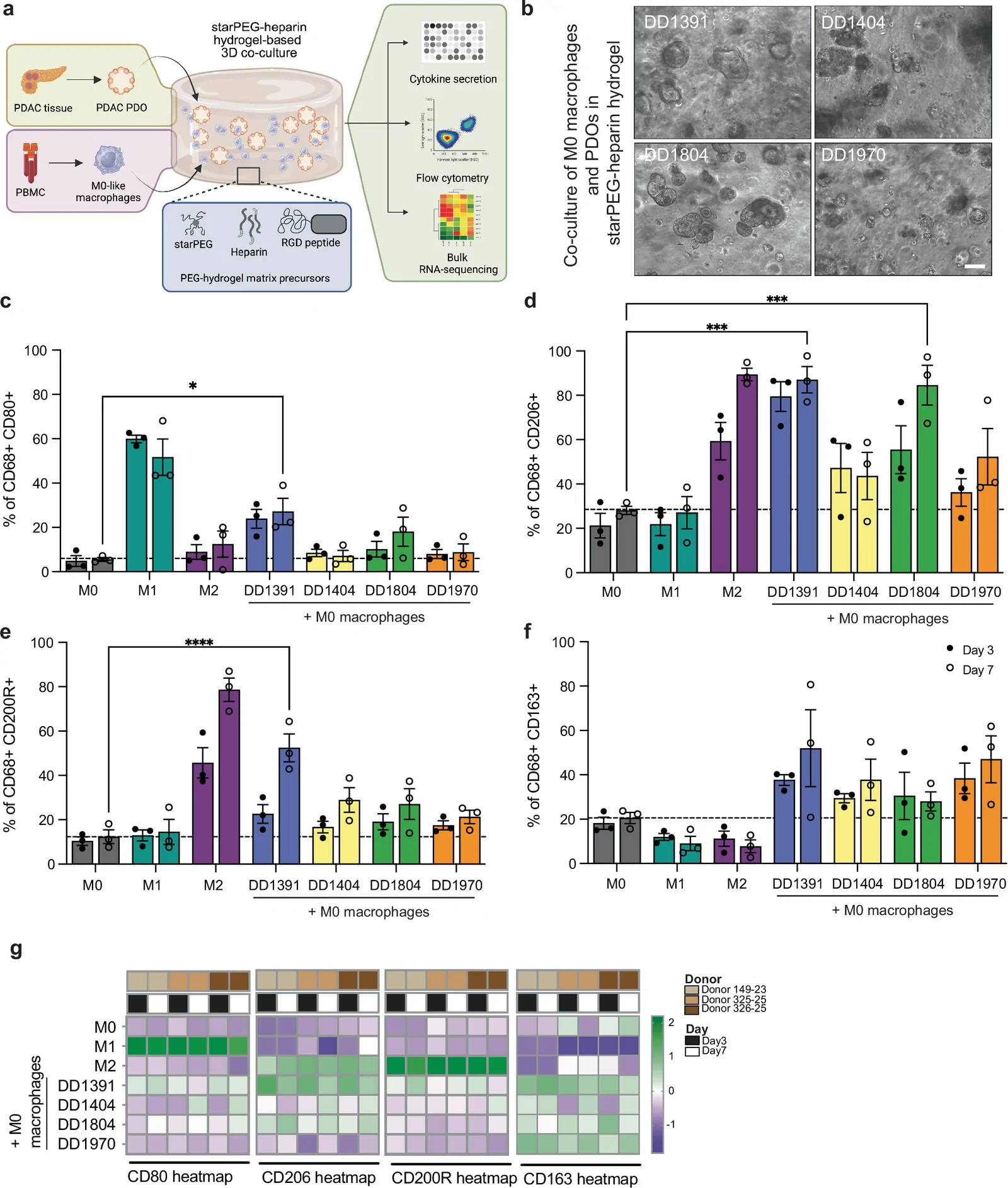
Macrophages show distinct polarization patterns with different PDAC PDO lines in a 3D co-culture platform. **a** Schematic overview of the starPEG-heparin hydrogel-based 3D co-culture system. **b** Representative brightfield images of starPEG-heparin hydrogels of the individual PDAC PDO lines co-cultured with M0 macrophages in co-culture medium. Scale bar: 130 µm. Flow cytometry analysis of in-vitro differentiated M0 and chemically induced M1-like and M2-like polarized macrophages used as positive controls and the starPEG-heparin hydrogel-based 3D co-cultures of PDAC PDOs and M0 macrophage donor 149-23 on day 3 (black dots) and day 7 (white dots) for the macrophage markers (**c**) CD80, (**d**) CD206, (**e**) CD200R and (**f**) CD163. The mean values and SEM of *n* = 3 independent replicates are displayed. ANOVA, *p < 0.05, ***p < 0.001, ****p < 0.0001. **g** Heatmap of flow cytometry-derived marker expression of in-vitro differentiated M0, M1-like, and M2-like macrophages, and M0 macrophages co-cultured with PDAC PDOs. Macrophages were derived from three independent donors (Donors 149–23, 325–25, and 326–25). Expression of the macrophage polarization markers CD80, CD206, CD200R, and CD163 was analyzed on day 3 and day 7. Z-transformed mean measurements of the double-positive population (e.g., CD68^+^CD206^+^) from *n* ≥ 2 independent biological replicates (Donor 149-23 *n* = 3; Donors 325-25 and 326-25 *n* = 2) are displayed.

To understand the impact of PDAC cells on macrophage polarization, we embedded macrophages sourced from three healthy donors (149-23, 325-25 and 326-25) together with PDAC PDOs in a co-culture at a 1:2 ratio using starPEG-heparin hydrogel and CCM (Fig. 5a). Brightfield imaging confirmed the distribution of both cell types within the starPEG-heparin hydrogels (Fig. 5b). Once co-cultured, macrophages no longer remained in their differentiated unpolarized state but instead adopted a polarized phenotype, as assessed by flow cytometry. Macrophages co-cultured with all PDOs exhibited overall low CD80 expression at day 3 and 7 (Fig. 5c, Supplementary Fig. S6a). At day 3, only DD1391 induced a modest increase in CD80⁺ macrophages, which by day 7 extended to also DD1804 and DD1404 (in 2 out of 3 donors), whereas DD1970 lacked CD80 upregulation at both time points. In contrast, M2-like CD206 expression was elevated in all co-cultures, except for donors 325-25 and 326-25 co-cultured with DD1970. Notably, CD206 levels were significantly higher on day 7 in macrophages co-cultured with DD1391 (*p* = 0.0011) and DD1804 (*p* = 0.0020) compared to M0, suggesting tumor-intrinsic macrophage polarization similar to an M2-like state and TAM phenotype, which both express this marker (Fig. 5d, Supplementary Fig. S6a). CD200R expression was similar to M0 macrophages at day 3 and showed a modest increase by day 7 across all co-cultures, with a more pronounced upregulation in the DD1391 co-culture at day 7 (*p* = 0.0001, Fig. 5e, Supplementary Fig. S6c). Interestingly, CD163, a known TAM marker in the cancer setting, was elevated in all tumor cell-conditioned co-cultures compared to mono-cultured M0 control and chemically induced M1 and M2 macrophages, without reaching statistical significance across conditions, likely reflecting the high inter-macrophage-donor variability (Fig. 5f, Supplementary Fig. S6d). Heatmap analysis across the three different donors at both time points (day 3 and day 7) revealed similar polarization patterns with reproducible CD80, CD206, and CD200R expression, whilst CD163 showed a more diverse polarization pattern. Macrophage-PDO co-cultures showed a distinct pattern compared to both chemically induced M1 and M2 controls, supporting a tumor cell-conditioned macrophage phenotype (Fig. 5g). The extent of PDO-induced macrophage polarization was consistent and independent of macrophage donor, pointing to tumor-intrinsic heterogeneity in shaping the immune TME.

Collectively, these results support that our defined 3D co-culture system captures the tumor-immune crosstalk, showing how PDAC organoids drive macrophages towards an intermediate M2-like immunosuppressive state.

### Transcriptomic analyses reveal TAM and M2 polarization signatures in PDO co-cultured macrophages

**Fig. 6:**
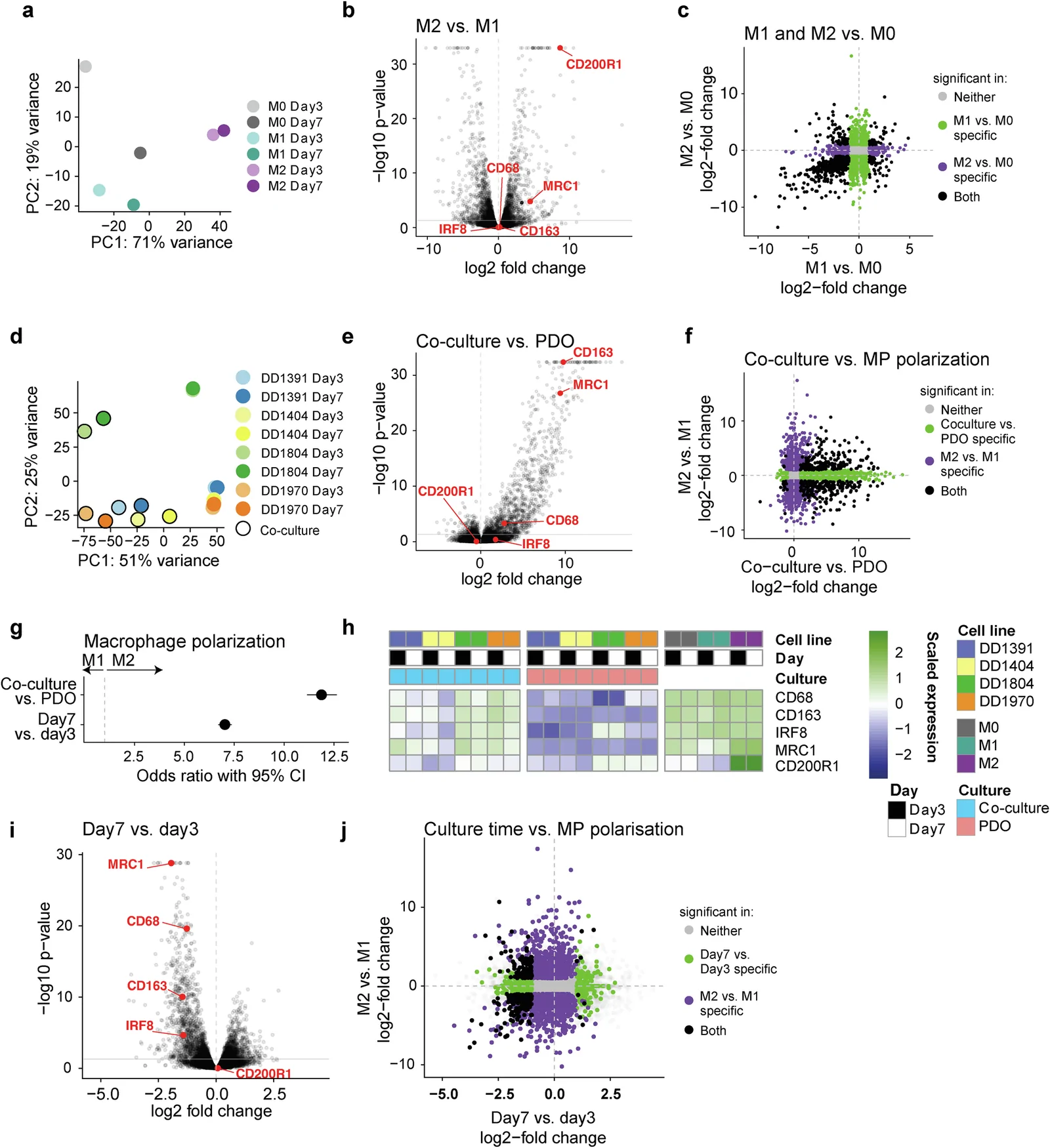
RNA sequencing of starPEG-heparin hydrogel co-culture and monocultures. **a** Principal component analysis (PCA) of in-vitro differentiated M0, M1-like, and M2-like macrophages. **b** Differential analysis of M2-like versus M1-like macrophages. *P*-values represent the adjusted *p*-values from the differential gene expression analysis. 5 marker genes are included that represent general macrophages (*CD68*), M1-like (*IRF8*), M2-like (*MRC1* & *CD200R1*), and tumor-associated macrophages (TAM; *CD163*). For visibility, the y-axis was capped at the highest highlighted gene, with all higher values plotted at this maximum. **c** Comparison of differential analyses between M2-like versus M0 and M1-like versus M0 including both day 3 and day 7 samples with correction. Significance thresholds refer to adjusted *p*-values of <0.05 and an absolute log2-fold change of 1. **d** PCA of PDOs and co-cultures. **e** Differential analysis of co-cultures versus PDOs. *P*-values represent the adjusted *p*-values. The same 5 marker genes are included. For visibility, the y-axis was capped at the highest highlighted gene, with all higher values plotted at this maximum. **f** Comparison of differential analyses between co-cultures versus PDOs and M2-like and M1-like macrophages, including both day 3 and day 7 samples with correction. Significance thresholds refer to adjusted *p*-values of <0.05 and an absolute log2-fold change of 1. **g** Contingency table analysis for odds ratios of M2-like macrophages being enriched in the co-cultures, given their balance to M1 in the co-culture-independent polarization. Given are the odds ratio with a 95% confidence interval and the *p*-values of an X^2^ test. The groups are defined by the significantly deregulated genes, where significance thresholds refer to adjusted *p*-values of <0.05 and an absolute log2-fold change of 1. **h** Heatmap of variance-stabilized gene expression counts for marker genes in all investigated samples. Relative counts are scaled by row. The same 5 marker genes are included. **i** Differential analysis of co-cultures of day 7 versus day 3. *P*-values represent the adjusted *p*-values. The same 5 marker genes are included that represent general macrophages (*CD68*), M1-like (*IRF8*), M2-like (*MRC1* & *CD200R1*), and tumor-associated macrophages (TAM; *CD163*). For visibility, the y-axis was capped at the highest highlighted gene, with all higher values plotted at this maximum. **j** Comparison of differential analyses between co-cultures of day 7 versus day 3 and co-cultures versus PDOs, including both day 3 and day 7 samples with correction. Significance thresholds refer to adjusted *p*-values of <0.05 and an absolute log2-fold change of 1.

To generalize our findings of the cell-cell communication between the PDAC PDOs and macrophages, we performed transcriptomic analysis across our models. Principal Component Analysis (PCA) of macrophage transcripts (Fig. 6a) and hierarchical clustering (Supplementary Fig. S7a) showed that they primarily separate by line and secondarily by the culture period. Differential analysis between M1 and M0 macrophages confirmed *IRF8* as a significantly upregulated marker gene, while the marker gene for M2 polarization, *CD200R1* was significantly downregulated (Supplementary Fig. S7b). Differential analysis between M2 and M0 showed a significant upregulation of the marker genes for M2 polarization markers, *MRC1* and *CD200R1*, yet the M1 marker was also significantly upregulated (Supplementary Fig. S7c). Comparing M2- and M1-polarized macrophages (Fig. 6b), both *MRC1* and *CD200R1* were significantly upregulated. However, the M1 polarization marker *IRF8* and the TAM marker *CD163* were unaltered in this comparison. Both polarizations shared 376 downregulated genes in comparison to M0 (Fig. 6c), yet only 43 upregulated genes, while 105 genes were regulated in opposite directions. We then integrated the derived gene expression changes into the analysis of the co-cultures. The samples primarily separated by cell line, then culture type, and lastly culturing time (Fig. 6d, Supplementary Figs. S7d). Differential analysis between co-culture and PDOs revealed significant upregulation of the M2 marker *MRC1* and *CD163*, while the M1 polarization marker *IRF8* and the other M2 marker *CD200R1* were unchanged in the transcriptome (Fig. 6e). Together, this indicated polarization towards M2 and a change of macrophages towards a tumor-associated phenotype. To generalize beyond known marker genes, we took advantage of the derived gene expression changes of the experimentally polarized macrophages, M2 vs M1 (Fig. 6f). Indeed, there were 423 shared significantly altered genes dependent on co-culture and macrophage polarization, while 1602 were altered only in the macrophages and 702 only in the co-cultures. This allowed assessment of polarization, asking whether more deregulated genes in the co-cultures fall into the M2 polarization group given the comparison with the gene group that was independent of the co-culture (Fig. 6g). Indeed, we observed a significant odds ratio of 11.9 (CI 11.2–12.6, *p* < 10–15, X^2^ test) in favor of M2 polarization. Analogously, we asked whether a comparison of co-cultures between day 7 and day 3 further followed this trajectory. Indeed, also in this comparison we observed a significant odds ratio of 6.1 (CI 5.8–6.3, *p*-<10–15, X^2^ test) in favor of continued development of M2 polarization from day 3 to day 7. Yet, when assessing the marker genes across all samples, it indicated complex and cell line-specific relationships with polarization (Fig. 6h). Expanding the set to a wider range of genes associated with macrophage polarization (Supplementary Fig. S7e), consistent expression patterns in the co-cultures lead to close clustering proximity of the TAM marker *CD163* and the M2 marker *MRC1*. Interestingly, other genes join the cluster (*TREM2*, *SIGLEC10*, *CNRIP1*, *MMP9*), which have previously been associated with the TAM phenotype. To assess the dependence on culturing time, we performed differential analysis between day 7 and day 3, which shows a general significant downregulation of all macrophage markers, except for the M2 marker *CD200R1* (Fig. 6i) and differential macrophage polarization genes in general (Fig. 6j). This indicates a relative reduction of macrophages in comparison to PDOs, likely due to continuous growth of the PDOs in co-culture in comparison to the increasingly M2 polarizing macrophages.

### Individual co-culture cell lines show consistent macrophage polarization behaviors

**Fig. 7:**
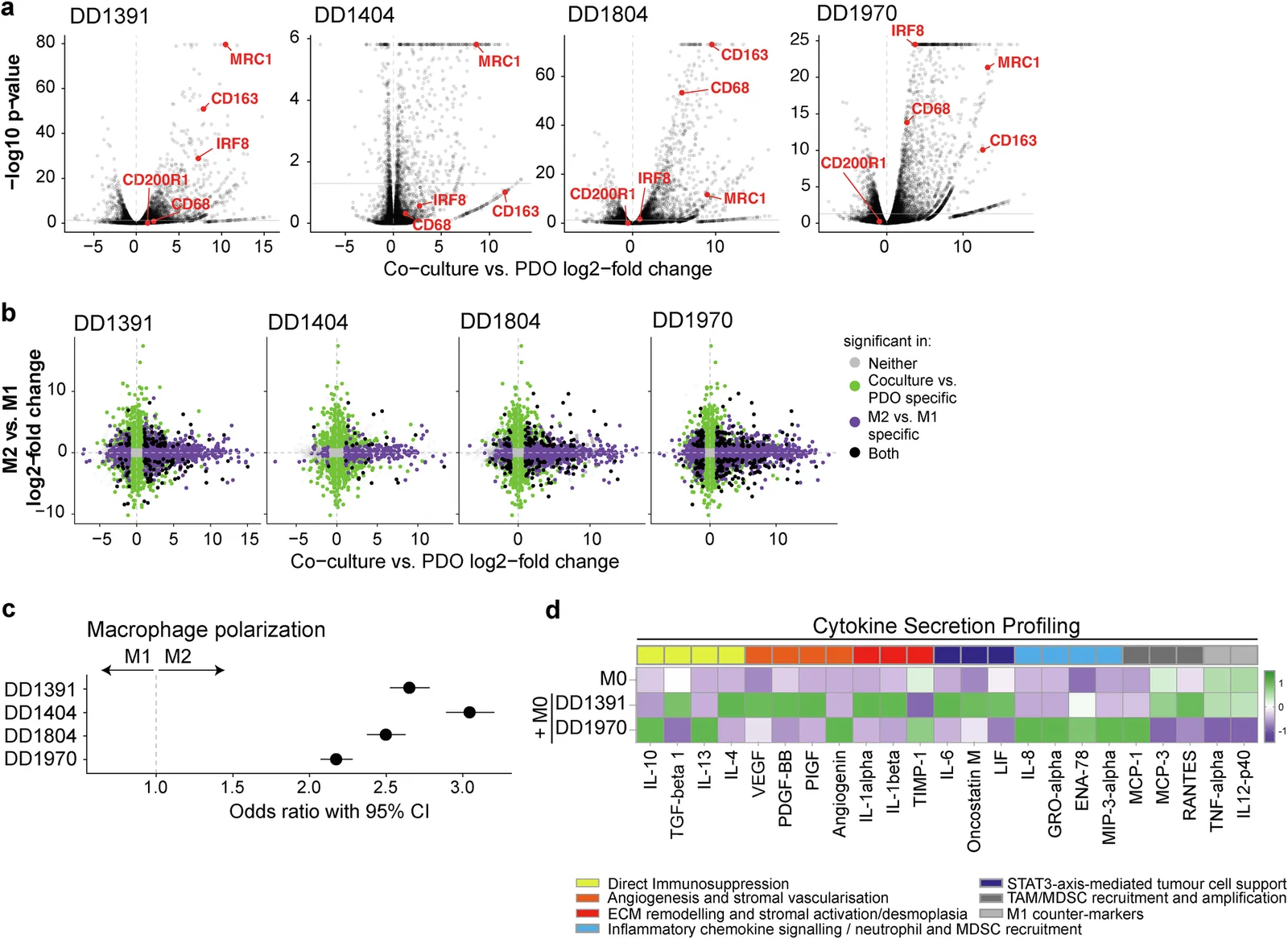
RNA sequencing and cytokine secretion profiling of starPEG-heparin hydrogels for individual co-culture cell lines. **a** Differential analysis of co-cultures versus PDOs for individual cell lines. *P*-values represent the adjusted *p*-values from the differential gene expression analysis. 5 marker genes are included that represent general macrophages (*CD68*), M1-like (*IRF8*), M2-like (*MRC1* & *CD200R1*), and tumor-associated macrophages (TAM; *CD163*). For visibility, the y-axis was capped at the highest highlighted gene, with all higher values plotted at this maximum. **b** Comparison of differential analyses between co-cultures versus PDOs and M2-like and M1-like macrophages for individual cell lines, including both day 3 and day 7 samples with correction. Significance thresholds refer to adjusted *p*-values of <0.05 and an absolute log2-fold change of 1. **c** Contingency table analysis for odds ratios of M2-like macrophages being enriched in the co-cultures, given their balance to M1 in the co-culture-independent polarization. Given is the odds ratio with a 95% confidence interval and the *p*-values of a X^2^ test. The groups are defined by the significantly deregulated genes, where significance thresholds refer to adjusted *p*-values of <0.05 and an absolute log2-fold change of 1. **d** Heatmap of soluble cytokines in the supernatants collected on day 7 from either M0 macrophage monocultures or co-cultures with PDAC PDOs DD1391 and DD1970 with M0 macrophages embedded in starPEG-heparin hydrogel matrix. Values were normalized to the M0 macrophage monoculture control and are shown as z-scores. Data depicted from a single independent experiment (*n* = 1).

Given the phenotypic and molecular heterogeneity of the PDO lines, we analyzed each line individually to delineate shared versus PDO-specific macrophage polarization patterns (Fig. 7a). Of note, macrophage-induced changes in PDOs were not examined in this study. Despite inter-PDO variability, co-culture elicited a broad consistent response: PDO co-cultures from all four patients showed a significant upregulation of the M2 marker *MRC1*. The M2-like marker *CD163* was significantly upregulated in three lines and showed a non-significant trend towards upregulation in DD1404, which displayed the lowest statistical power and smallest log2 fold-changes, likely reflecting its comparatively low macrophage content in co-culture (Fig. 6h). The M2 polarization marker *CD200R1* was unchanged or undetected in all samples (Fig. 7a) whilst *IRF8*, a transcription factor classically linked to M1 activation but also reported in TAMs in the context of T-cell exhaustion, was significantly upregulated in DD1391 and DD1970. We therefore asked whether we may indeed have different polarization trajectories in the different cultures and investigated significantly altered genes with respect to M2 versus M1 comparison and co-culture versus PDO monocultures. As expected from the reduced sample sizes relative to the joint analysis (Fig. 6f, g), PDO specific analyses yielded fewer shared differentially expressed genes (Fig. 7b). Nevertheless, the derived odds ratios consistently favored M2 over M1 polarization in all four PDO co-cultures (Fig. 7c). We therefore concluded that based on the transcriptomes of the experimentally polarized macrophages, the co-cultured macrophages aligned more strongly with an M2-like polarization more than M1-like polarization state. This polarization continued between day 3 and day 7. In addition, the M2-like marker *CD163*, which was not represented by the experimentally polarized macrophages, was increasingly expressed as well, which indicated that additional tumor-mediated changes to macrophages can be induced by the co-culture. This was consistent with the expression of the macrophage markers using mIHC. Overall, our 3D co-culture system of the patient-matched organoids with the primary tissue demonstrates that we can recreate a pronounced immunosuppressive TME of PDAC ex vivo, which may be leveraged to investigate macrophage–tumor interactions and therapeutic modulation.

### Cytokine secretome profiling reveals PDO-specific induced macrophage function

To functionally characterize the soluble mediators underlying the surface marker- and transcriptome-based macrophage polarization, we performed multiplex cytokine profiling of supernatants from M0 macrophages alone (M0) and two selected PDO-macrophage co-cultures (DD1391 + M0 and DD1970 + M0) after 7 days using a membrane-based protein array (Fig. 7d). Compared to M0 macrophages alone, both PDO co-cultures showed an altered expression of immunosuppressive and pro-tumorigenic factors, notably with cytokine profiles distinct from the macrophage polarization state. M1-associated cytokines TNF-alpha and IL-12 p40 were reduced in both co-cultures compared to M0 alone, consistent with the loss of an M1-like signature already observed by flow cytometry and transcriptomic analysis. DD1391 co-cultured macrophages showed elevated secretion of TGF-beta 1, VEGF, PDGF-BB, IL-6, oncostatin M, LIF, and IL-4, reflecting a more dominant M2-like/TAM-like phenotype with combined immunosuppressive, pro-angiogenic, and tumor-supporting features. Notably, the elevated IL-4 and TGF-beta 1 levels in the 3D co-culture were consistent with the marked CD206 surface expression observed by flow cytometry, as both cytokines are established inducers of the mannose receptor. By contrast, DD1970 co-cultured macrophages displayed a distinct, chemokine-driven signature characterized by elevated IL-10, MCP-1 (CCL2), GRO-alpha (CXCL1), IL-8 (CXCL8), ENA-78 (CXCL5), MIP-3-alpha (CCL20), angiogenin, and TIMP-1, consistent with an IL-10-dominated immunosuppressive phenotype and recruitment-associated chemokine signaling. Since the M0 macrophage input was identical across both 3D co-cultures, these divergent cytokine signatures most likely reflect differential macrophage education by tumor-intrinsic cues, although PDO-specific contributions to the secretome cannot be excluded. These findings functionally corroborate the inter-PDO heterogeneity observed at the surface marker and transcriptional level and indicate that PDO-driven macrophage polarization comprises multiple, biologically coherent yet distinct co-culture secretory profiles and signatures.

## Discussion

The desmoplastic and immunosuppressive TME of PDAC is a major contributor to its aggressive behavior and resistance to chemo- and immunotherapies^2,32^. TAMs are among the most abundant and prognostically relevant immune cell populations in PDAC, promoting immune evasion, therapy resistance, and metastasis^17^. Dissecting their crosstalk with cancer cells has been limited by the lack of physiologically relevant models that preserve cell functions, 3D architecture, and the complexity of the TME. Here, we established a fully defined 3D co-culture model for mechanistic interrogation of tumor-macrophage interactions in PDAC using a starPEG-heparin hydrogel matrix. Existing human tumor-macrophage models commonly use tumor cell-conditioned medium or exogenous cytokines to induce TAM-like states^30,31^, but they lack stromal architecture, mechanobiological cues, and spatial organization. More complex systems, such as macrophage-augmented organoids that closely mimic in-vivo viral infections of the liver, illustrate the value of incorporating myeloid components into the organoids^33^. Tunable hydrogel platforms now allow systematic interrogation of TME biophysics^34^. StarPEG-heparin hydrogels provide highly reproducible and fully defined control of matrix stiffness and biochemical composition, thus overcoming the variation seen in animal-derived matrices. Matrix mechanics shape not only tumor behavior and treatment response, such as reversible chemoresistance of PDAC PDOs in stiff, fibrosis-like matrices^24,35,36^, but also regulate macrophage viability, recruitment, and phenotype^37,38^. In this context, the starPEG-heparin hydrogel used herein provides a suitable mechanobiological niche for examining macrophage education by PDAC cells in a stromal-mimetic setting^39^.

Multiplex IHC profiling of primary PDAC tissues confirmed that M2-like and TAM-like macrophages are the predominant intratumoral population, with their highest density in the stromal compartment, consistent with single-cell and spatial transcriptomic studies describing that immunosuppressive desmoplastic niches correlate with poor prognosis^40,41^. A meta-analysis by McGuigan and colleagues reported that infiltration of M2-like CD163^+^ macrophages, rather than total CD68^+^ macrophage density, associates with poor outcome, indicating that macrophage polarization is a stronger prognostic factor than abundance^42^. Sivakumar and colleagues further identified myeloid-enriched PDAC subtypes, characterized by high infiltration of CD163^+^ TAMs and low CD8^+^ T-cell abundance, that correlated with poorer survival, which was validated in an independent cohort (APACT trial). This underscores the prognostic and therapeutic relevance of the tumor-TAM crosstalk^43^.

Consistent with these observations, across multiple PDO lines, co-cultured M0 macrophages consistently acquired an M2-like and TAM-like phenotype, with increased CD163 and CD206 expression and induction of TAM-associated transcriptional signatures^44–46^. Transcriptomic analysis revealed upregulation of TAM-associated markers such as *TREM2* and *SIGLEC10* that are distinct from IL-4-induced M2 macrophages. Molgora and colleagues showed that TREM2-expressing TAMs promote tumor growth, thereby limiting the efficacy of anti-PD-1 checkpoint immunotherapy. The genetic deletion or antibody-mediated inhibition of TREM2 rewired the myeloid compartment towards an immunostimulatory phenotype with increased T cell responses to anti-PD-1 therapy, highlighting TREM2 as a promising target in immuno-oncology ^47^. SIGLEC10, which can also be highly expressed on TAMs, can interact with CD24 on tumor cells, thereby eliciting a ‘don’t-eat-me’ signal that allows the tumor to evade macrophage-mediated phagocytosis. The disruption of the CD24-SIGLEC10 axis by blockade or genetic deletion of SIGLEC10 or CD24 can augment clearance of CD24-expressing tumors and increase survival^48^. While mechanistic dissection of these pathways is beyond the scope of the present study, our platform could serve as a useful system in which distinct signaling cascades could be perturbed to test the causal contribution of specific proteins to the immunosuppressive TAM phenotype and to evaluate macrophage-directed interventions.

Cytokine secretome profiling of two selected PDO–macrophage co-cultures further corroborated our findings at the protein level and revealed distinct macrophage signatures in response to different PDAC PDOs. The limited abundance of M1-like macrophages in our 3D model may reflect the combined effects of PDAC-secreted factors and matrix stiffness, both known to impact inflammatory macrophage polarization. Because the cytokine profiling was performed only on two representative PDO lines, the resulting signatures should be regarded as proof-of-concept observations rather than definitive evidence of generalizable macrophage subclasses. Validation across a larger panel of PDOs and macrophage donors will be required to determine whether these subclasses represent reproducible, discrete TAM states in PDAC. Furthermore, as the cytokine profiling array measured secreted proteins from the PDAC PDO-macrophage co-culture, the measured cytokines reflect the combined output of both cell types and therefore cannot be attributed specifically to macrophage-specific signals from the overall secretome.

In addition to soluble tumor-derived cues, mechanical properties of the surrounding matrix have also been implicated in macrophage polarization. Varying hydrogel stiffness (i.e., crosslinking density of starPEG to heparin) within the tested range did not significantly alter the main macrophage polarization patterns in our co-culture system. This suggests that, under the conditions examined here, PDO-derived cues were the dominant drivers of macrophage polarization^37^. This is contrary to previous studies of the mechanosensitivity of macrophages. Substrate stiffness has been shown to tune macrophage phenotype and function, for example through the mechanically activated ion channel Piezo1^49^, and via stiffness-dependent shifts in polarization and migration^50^. The starPEG-heparin hydrogel used here has different adhesive and growth-factor-sequestering properties compared to the collagen- or fibronectin-rich substrates in which most macrophages are usually tested. In addition, the tumor-derived secretome may override the comparatively subtle mechanical cues within the measured stiffness here, particularly in the absence of other important mechanoresponsive stromal lineages such as fibroblasts, which are not included in the present platform. A broader range of matrix stiffness and culture composition will therefore be needed to fully resolve the contribution of mechanical cues to macrophage polarization in this system.

The deliberate restriction of our platform to PDO-macrophage interactions offers a complementary advantage over more complex multicellular 3D models. The ability to selectively model and perturb defined components provides a powerful framework to identify causal interactions, uncover macrophage-specific therapeutic vulnerabilities, and generate hypotheses that can subsequently be tested in multicellular systems.

Of note, interpretation of the data presented here distinguishes different levels of biological complexity: PDO-specific (tumor-intrinsic) effects that originate from the cancer cells themselves, co-culture-specific effects that emerge from reciprocal PDO-macrophage interactions, and patient-specific effects that reflect the in-vivo complexity of an individual patient’s TME. Our co-culture model provides a proof-of-concept for reconstructing the patient-specific complexity using PDOs while retaining the ability to selectively tune and analyze the isolated cancer-cell–macrophage interactions. These findings warrant further studies to enhance physiological relevance by integrating additional stromal and immune constituents, such as CAFs, T-cells, and endothelial cells, that also shape macrophage behavior in-vivo. Furthermore, macrophages were derived from healthy donors for our 3D co-cultures. Incorporating donor-to-donor variation or patient-derived autologous macrophages will help to address the inter-individual variability in macrophage education. It will also allow exploration of differences between monocyte-derived and tissue-resident macrophage populations, which remain difficult to capture due to availability and technical constraints. Future work may examine how macrophages influence organoid phenotype, transcriptional programs related to matrix remodeling, stemness, epithelial states, and treatment response, building on the co-culture framework established here. The heterogeneity in macrophage education observed here underscores the translational potential of this platform for precision oncology. As organoid drug profiling has been shown to capture clinically meaningful, patient-specific therapeutic vulnerabilities in PDAC^51^, extending this concept to tumor-macrophage co-culture could allow patient stratification according to the immunosuppressive TAM state imposed by their tumor cells. The defined, two-cell format is also well suited for ex vivo testing of immunomodulatory interventions that target distinct aspects of macrophage biology, including TAM reprogramming, inhibition of monocyte recruitment (e.g., CCR2 inhibitors), CSF1R blockade, and ‘don’t-eat-me’ checkpoint blockade (anti-CD47), in a patient-specific context. This approach could enable functional preselection of candidate strategies before evaluation in more complex or in-vivo systems, in line with emerging macrophage-targeting therapeutic approaches^52^. Although these applications remain prospective and will require validation in larger, clinically well-annotated cohorts, they may illustrate how a scalable, fully defined organoid-macrophage co-culture could contribute to biomarker discovery, patient stratification, and the personalized evaluation of immune-directed therapies in PDAC.

In conclusion, this work presents a defined 3D PDAC organoid–macrophage co-culture model as a proof-of-concept that recapitulates integral immunosuppressive features of the pancreatic TME. Our 3D co-culture system supports the targeted mechanistic dissection of tumor-driven macrophage education in a minimalistic, fully defined starPEG-heparin hydrogel. By isolating human PDO-macrophage interactions from the broader multicellular microenvironment, this platform provides a defined and experimentally tractable tool to dissect tumor–myeloid crosstalk and uncover candidate mechanisms of macrophage education. More importantly, beyond mechanistic insight, we view this 3D platform as a complementary, human-relevant platform that enables accessible and cost-efficient hypothesis testing and preselection of macrophage-directed therapies before evaluation in living systems and resource-intensive settings. Future work will focus on the application of the 3D platform for translational research to identify potential therapeutic targets, enhance anti-tumoral immunity, and support personalized treatment strategies.

## Methods

### Patient cohort

All procedures involving human participants were conducted in accordance with the ethical standards of the institutional and national regulations, and with the Declaration of Helsinki. Preoperative blood samples and PDAC tissue biopsies for organoid generation were collected from patients diagnosed with resectable PDAC at the University Hospital of Dresden between 2022 and 2023 (Supplementary Table S1). All PDAC patients provided written informed consent, and the study was approved by the Ethics Committee of the TU Dresden (EK76032013; EK366092024). All tissue samples were reviewed by board-certified pathologists to confirm the presence of PDAC according to the WHO classification. Human peripheral blood mononuclear cells (PBMCs) were obtained from PDAC patients for the optimization of the co-culture conditions. PBMCs from G-CSF-mobilized leukapheresis of healthy donors, which was undertaken at the Bone Marrow Transplantation Center of the Carl Gustav Carus University Hospital, Dresden, Germany, under a study protocol approved by the Ethics Committee of the TU Dresden (EK127042009) and with written informed consent from all donors, were used for co-culture experiments. Leukapheresis products were transferred to the Leibniz Institute of Polymer Research Dresden, Max Bergmann Center of Biomaterials, Dresden, Germany, where they were washed and processed according to the CD34 MicroBead Kit UltraPure (Miltenyi Biotec). During magnetic-activated cell sorting of CD34-expressing hematopoietic stem and progenitor cells, the CD34-negative flow-through, containing monocytes, macrophages, and other blood cells, was separately collected. Then, the CD34-negative cell collection was biobanked at 50×10^6^ to 100×10^6^ per 1 ml vial in a custom, step-wise freezing workflow including 50% (v/v) CryoStor CS10 (Stem Cell Technologies), 10% (v/v) CryoSure-DMSO (WAK-Chemie Medical), and 5% (v/v) human serum albumin (HSA, Baxalta) in PBS, and stored in a liquid nitrogen tank until further use. These were used for monocyte isolation, macrophage differentiation, and for co-culture with PDAC PDOs.

### Isolation and differentiation of monocytes into macrophages

Monocytes (CD14^+^) were isolated from biobanked PBMCs via negative magnetic bead selection using the Pan Monocyte Isolation Kit (Miltenyi Biotec, Bergisch Gladbach, Germany) according to the manufacturer’s instructions. Cell viability was assessed by trypan blue exclusion prior to culture. Purified monocytes were seeded on NUNC™ UpCell™ plates (Thermo Fisher, Waltham, US) and maintained in macrophage medium consisting of RPMI1640 (Thermo Fisher, Waltham, US) supplemented with human serum (10% v/v, Sigma-Aldrich, St. Louis, US), M-CSF (25 ng/ml, Miltenyi Biotec, Bergisch Gladbach, Germany), and Penicillin/Streptomycin (1% v/v). To generate M0 macrophages, monocytes were treated with M-CSF and on day 6 either subjected to LPS (100 ng/ml, Sigma-Aldrich, St. Louis, US) and IFN-γ (10 ng/ml, Gibco, Waltham, US) for M1 macrophage polarization, or to IL-4 (20 ng/ml, Miltenyi Biotec, Bergisch Gladbach, Germany) for M2 macrophage polarization.

### Multiplex immunohistochemistry

Formalin-fixed, paraffin-embedded (FFPE) tumor sections from nine PDAC patients were stained using the Opal multiplex technology (Akoya Biosciences) on the Ventana Discovery Ultra platform (Ventana Medical Systems, RRID:SCR_021254). Tissue slides underwent deparaffinization, rehydration, and heat-induced antigen retrieval at 95°C in Cell Conditioning Solution (CC1, pH 9; Ventana Medical Systems). Primary antibodies (Supplementary Table S2) were applied sequentially, followed by horseradish peroxidase (HRP)-conjugated OmniMap secondary antibodies (Ventana Medical Systems). Opal TSA fluorophores (Akoya Biosciences) were then introduced, enabling covalent binding to tyramide residues and stable fluorescence emission. Antibody stripping was performed after each cycle using CC2 buffer (pH 6; Ventana Medical Systems) at 100°C to allow sequential staining of up to six markers. After completing all cycles, nuclei were counterstained with DAPI (Merck/Sigma-Aldrich), and slides were mounted using Fluoromount-G (SouthernBiotech). Afterwards, whole-slide scans were acquired using PhenoImager HT 2.0 (Akoya Biosciences). Regions of interest (ROIs) were defined using Phenochart software (Akoya Biosciences, RRID:SCR_019156), followed by multispectral imaging (MSI) at 200x magnification. Spectral unmixing was performed in inForm software (Akoya Biosciences, RRID:SCR_019155), and multi-channel TIFF files were generated for downstream analysis. Finally, the images were processed in QuPath software following an established pipeline (https://qupath.readthedocs.io/, RRID:SCR_018257). Initially, 80–100 randomly selected images were used to train a pixel classifier for segmenting tumor and stromal regions. Using the QuPath extension StarDist, automated cell detection was executed^53,54^, followed by training object classifiers to identify specific cell types based on marker expression. A validation set of images was used to refine classification accuracy before applying the final pipeline, integrating pixel classification, cell detection, and object classification across all images.

### PDAC organoid generation from tissue specimens

Tumor biopsies were washed, minced into small pieces (<1 mm^3^), and enzymatically digested with DispaseII (1.2 mg/ml, Roche, Basel, Switzerland) and CollagenaseII (0.625 mg/ml, Sigma-Aldrich, St. Louis, US) in DMEM/F12+++ medium supplemented with 1xHEPES, 1xPen/Strep and 1xGlutaMAX (all Invitrogen, Waltham, US) at 37 °C as previously described^10^, and cultivated in human PDAC organoid medium DMEM/F12+++ supplemented with Wnt3a-conditioned medium (50% v/v), noggin-conditioned medium (10% v/v), RSPO1-conditioned medium (10% v/v), B27 (1x, Invitrogen, Waltham, US), nicotinamide (10 mM, Sigma-Aldrich, St. Louis, US), gastrin (1 µM, Sigma-Aldrich, St. Louis, US), N-acetyl-L-cysteine (1.25 mM, Sigma-Aldrich, St. Louis, US), primocin (100 µg/ml, InvivoGen, San Diego, US), recombinant murine epidermal growth factor (50 ng/ml, Invitrogen, Waltham, US), recombinant human fibroblast growth factor 10 (100 ng/ml, PeproTech, Waltham, US), A-83-01 (0.5 μM, Tocris Bioscience, Bristol, UK), and N2 (1x, Invitrogen, Waltham, US)^10^. The organoids were always maintained at 37 °C and 5% (v/v) CO_2_. PDAC organoid lines were routinely tested for mycoplasma using MycoStrip™ DETECTION Assay (InvivoGen, San Diego, US).

### Cell viability and caspase 3/7 assays

For all viability-related experiments, PDAC organoids and macrophages were plated in 96-well plates and assessed over a 7day period. Cell viability was measured using the CellTiter-Glo® Luminescent Cell Viability Assay (Promega, Madison, US) according to the manufacturer’s instructions. Luminescence was recorded using the Varioskan Lux (Thermo Fisher Scientific, Waltham, US) with an integration time of 500 ms. Caspase-3/7 activity was quantified using the Caspase-Glo® 3/7 Assay (Promega, Madison, US), following the manufacturer’s protocol, and luminescence was measured with a 500 ms integration time. For both assays, measurements were normalized to the first day of culture (day 1) and depicted as fold change.

### StarPEG-heparin hydrogel setup for 3D mono- and co-culture

Matrigel-embedded organoids were mechanically dissociated using a fire-polished glass Pasteur pipette. A relative cell number was achieved by digesting PDOs into single cells using TrypLE^TM^ Express (Thermo Fisher, Waltham, US). Macrophages were incubated with Dulbecco’s PBS for 15 min at RT on a rocker and detached using a 1% BSA-coated pipette. Cell viability was assessed with trypan blue. PDAC organoids (3 × 10^4^ cells/ml) and M0 macrophages (6 × 10^4^ cells/ml) were mixed in a ratio of 1:2 for co-cultures, based on established organoid–immune cell co-culture models in which this ratio balances sufficient macrophage numbers for reliable functional readouts with avoidance of non-specific cytotoxicity at higher ratios^55,56^; notable that seeding ratios in tumor cell-macrophage co-culture models vary across the literature (1:1 to 1:10), reflecting the absence of a universal standard. Monocultures of PDAC organoids and M0 macrophages, along with M1- and M2-polarized macrophages, were used as controls. Protease-sensitive starPEG-heparin hydrogels for cell embedding were composed of star-shaped poly(ethylene glycol)-peptide conjugates (starPEG, MW 15,000 Da). For this, heparin was first dissolved in Dulbecco’s PBS (pH 7.4) and was covalently functionalized with 2 moles of thiol-containing RGDSP (MW 990 Da) per mole of heparin via a Michael addition reaction. Then, the cells were suspended in the hydrogel precursor solution containing RGDSP and heparin. This mixture was then combined with a secondary hydrogel precursor solution of starPEG dissolved in Dulbecco’s PBS (pH 7.4). The two components were mixed at an equimolar ratio, corresponding to a crosslinking ^26^ratio of 1 (i.e., as defined as *γ*=1 in prior studies)^26^. The cell-containing starPEG-heparin hydrogels were cast onto sterile, Sigmacote-coated glass slides (Sigma-Aldrich, St. Louis, US). Upon gelation (5 min at RT), the drops were transferred into NUNC™ UpCell™ cell culture plates containing the co-culture medium (CCM) consisting of RPMI supplemented with Wnt3a-conditioned medium (20.5% v/v), noggin-conditioned medium (5% v/v), RSPO1-conditioned medium (5% v/v), B27 (0.5x, Invitrogen, Waltham, US), DMEM/F12+++ (14% v/v), human serum (1.5% v/v, Sigma-Aldrich, St. Louis, US) and Penicillin/Streptomycin (1%). Co-cultures were assessed at day 3 and day 7 to capture tumor–macrophage interaction, reflecting early polarization responses and sustained transcriptional reprogramming, respectively^57–59^. Medium was replaced with fresh CCM after 3 days.

### Flow cytometry

Two 10 µl hydrogel drops were pooled and treated with Collagenase type IA (1.85 mg/ml, Sigma-Aldrich, St. Louis, US) for cell recovery. After a maximum of 20 min at 37 °C, the cell suspension was centrifuged and washed with PBS. Samples were first stained with a viability dye followed by macrophage-specific markers, prior to fixation and permeabilization with eBioscience FOXP3/Transcription Factor Staining Buffer Set (Thermo Fisher Scientific, Waltham, US). FMO and isotype controls were used to guide gating (Supplementary Fig. S5b). Flow cytometry was performed using the LSR Fortessa™ flow cytometer (BD Bioscience, Franklin Lakes, US). Data were analyzed using FlowJov10.7.1 (BD Bioscience, Franklin Lakes, US).

### Cytokine profiling

For membrane-based cytokine profiling, supernatant of M0 macrophages and PDO co-cultures (DD1391 and DD1970) were harvested on day 7 and immediately stored at −80 °C until further analysis. Cytokine profiling was performed using the Human Cytokine Array C5 kit (RayBiotech, RRID:AB_10185250) according to the manufacturer’s instructions. Chemiluminescent membrane images were acquired using a Fusion FX imaging system (Vilber Lourmat, France). Densitometric quantification of protein array signal intensities was performed using the Protein Array Analyzer plugin in ImageJ^60^.

### Transcriptomic analysis

Differential gene expression analysis was performed using DESeq2^61^ (1.46.0) in R(4.4.1). PDO transcriptomes and co-cultures were combined into one object and macrophages into another. The DESeq2 design objects were generated, including a compensatory term for the culturing time, where cell lines or cultures were assessed, and a compensatory term for the cell line, where the culturing time was assessed. For PCA and hierarchical clustering, counts were first transformed with variance stabilization. For visualization of genes in a heatmap, gene expression was scaled by row. Differential gene expression analysis was performed with an adjusted *p*-value cut-off of 0.05 and an absolute log2-fold change of 1. For volcano plots showing differential gene expression, y-axes were capped at the highest highlighted genes, and every value above was collapsed at this maximum. The purpose was better visibility for the transcript population of interest given some outlier genes with very high significance levels. Odds ratios for macrophage polarization were derived from contingency tables showing the overlap of significant genes from the experimental polarization M2-like versus M1-like and the co-culture comparison to the PDOs or days 3 and 7 of culturing. Odds ratios with a 95% confidence interval were determined based on whether the proportion of M2-like genes in the co-culture versus M1-like genes in the co-culture is enriched as compared to the polarization genes that are independent of co-culture. For the associated testing statistics, an X^2^ test was performed. Marker genes were selected based on experimental data on protein levels and literature.

### Statistical analysis

Graphs and statistical analyses, including significance and *p*-values, were performed using GraphPad Prism v10.4.0 (RRID: SCR_002798). Unless otherwise stated, the data sets represent two independent experiments. Values were averaged, and standard deviation or, if applicable, standard error of the mean was calculated. A paired t-test was used for the mIHC analysis. An unpaired t-test or ANOVA was used for statistical analyses and is mentioned in the figure legends. The *p*-values are presented as follows: *p ≤ 0.05; **p ≤ 0.01; ***p ≤ 0.001; ****p ≤ 0.0001.

## Supplementary information

Revised Thyen, Sioson et al Supplementary Information_clean

## Data Availability

The data generated in this study are derived from patient samples and associated clinical information. Individual-level clinical data are not publicly available because they contain potentially identifying and sensitive patient information and because unrestricted public sharing is not permitted under the ethics approvals and informed consent obtained for this study. De-identified data supporting the findings of this study are available from the corresponding author upon reasonable request, subject to approval by the relevant institutional ethics/data access committee and completion of an appropriate data transfer agreement. Requests should be directed to franziska.baenke@ukdd.de. Processed, non-identifying data required to reproduce the main findings are provided in the manuscript and/or Supplementary Information. Sequencing data will be made available through controlled access where required by patient consent and data-protection regulations.

## Code availability

The data analysis files are available on Zenodo (https://doi.org/10.5281/zenodo.22111561).
