# Supplementary material for: A pancreatic cancer organoid-macrophage co-culture using starPEG-heparin hydrogel deciphers tumor-immune cell interactions
Source: NPJ Precis Oncol. 2026 Sep 5;10:343. doi: 10.1038/s41698-026-01678-6 (PMC13546286; doi:10.1038/s41698-026-01678-6)
Supplement: Supplementary file 1 [file 41698_2026_1678_MOESM1_ESM.pdf]

SUPPLEMENTARY INFORMATION

A pancreatic cancer organoid-macrophage co-culture using starPEG-heparin hydrogel deciphers tumor-immune cell interactions

Julius Thyen[1±*], Victor A. Sioson[1±*], Daniel Haak[1], Beatrix Jahnke[1], Maximilian Fusenig[2], Abdul M. Aftab[3], Antonia Stammberger[4], Sascha Brückmann[5], Heike Polster[1], Stefanie Huebner[1], Verena J. Kast[2], Manuel Pfeifer[6], Rebecca Prause[7,8], Vivian Mittné[1], Carolin Beer[1,8], Malin H. Reinhart[1], Adrian M. Seifert[1,8,12], Lena Seifert[1,8,9,12], Martin Bornhäuser[8,10], Carsten Werner[2,11], Marc Schmitz[4,8,12], Daniela E. Aust[5], Jürgen Weitz[1,8], Daniela Lössner[2,13,14], Daniel E. Stange[1,8,12], Anna R. Poetsch[3,8,15,16,17#*], Mathieu Pecqueux[1,7#*], Franziska Baenke[1,7,11#*]

SUPPLEMENTARY METHODS

Isolation of human peripheral blood mononuclear cells (PBMCs)

PBMCs were isolated from heparinized whole blood using the SepMate™ isolation protocol (STEMCELL Technologies, Vancouver, Canada). The PBMC pellets were treated with ACK lysis buffer (Thermo Fisher Scientific, Waltham, US) for the removal of erythrocytes and stained with trypan blue (Thermo Fisher Scientific, Waltham, US) for assessing cell viability. Cells were cryopreserved using cell culture freezing medium (Gibco, Waltham, US).

Immunohistochemistry staining

Organoids were first harvested from Matrigel using Cell Recovery Solution (Corning, New York, US) prior to being fixated with 4% formaldehyde and embedded into paraffin. Immunohistochemical analysis for the samples was performed according to

standard protocols using hematoxylin and eosin (H&E) staining and primary antibodies: CK19 (Abcam, RRID:AB_445429; Dilution 1:100) and TP53 (Cell Signaling Technology, RRID:AB_331743; Dilution 1:200) (**Supplementary Table S2**). Antigen retrieval was performed by heating the samples in 0.01M citrate buffer, pH6. The Signal Stain Detection Boost IHC/HRP mouse (Cell Signaling Technology, RRID:AB_10547893) was used for detection. Brightfield images were taken using the EVOS FL Auto microscope (Invitrogen, Waltham, US, RRID:SCR_026039).

DNA isolation and WGS sequencing

Organoids from 5-10 wells of a 48-well plate were incubated for 30min on ice with Cell Recovery Solution (Corning, New York, US) to dissolve Matrigel. Following centrifugation, PDOs were washed in PBS, and gDNA was isolated using QIAamp DNA Mini Kit (Qiagen, Hilden, Germany) according to the manufacturer's instructions. WGS was performed, quality checked and processed by AZENTA (Leipzig Germany). Somatic small variant candidates were called from matched tumor-normal WGS BAM files using GATK Mutect2 against the Hg38 reference genome[56]. For each patient, PDO and tissue samples were called separately against the corresponding matched germline sample. The resulting tumor-normal Mutect2 VCF files were annotated with Ensembl Variant Effect Predictor[57] using the GRCh38 cache and converted to Mutation Annotation Format using vcf2maf[58]. For downstream visualization, variants were restricted to “coding non-silent” classes, including missense, nonsense, frameshift, in-frame insertion/deletion, splice-site, nonstop, and translation-start-site mutations. Oncoplots were generated from selected PDAC-associated genes to compare somatic alteration patterns between PDO and matched tissue samples.

Copy number alterations (CNAs) were identified using CNVkit (v0.9.13)[59]. For each PDO and primary tumor sample, the matched germline sample was used as the reference for copy number calling using the CNVkit WGS workflow. Integer copy number states were assigned using the threshold calling method (-m threshold) with a diploid baseline (--ploidy 2) and thresholds of -1.2, -0.25, 0.3, and 0.7, corresponding to deep deletion (CN=0), shallow deletion (CN=1), copy-number neutral (CN=2), shallow gain (CN=3), and amplification (CN≥4), respectively.

RNA isolation and Bulk RNA-sequencing

The isolation of RNA samples was performed by using the RNeasy Mini Kit according to manufacturer's instructions. Of note, RNA isolation of macrophages included ß-mercaptoethanol. RNA concentration was measured using the NanoVue plus spectrophotometer (Thermo Fisher, Waltham, US).

RNA-Seq libraries were prepared from 100ng of high-quality RNA (RIN>7) according to the Illumina stranded total RNA Prep, Ligation with Ribo-Zero Plus (Illumina, San Diego, CA, USA) protocol and sequenced 2x74bp PE on an Illumina NextSeq2000 platform (RRID:SCR_023614) to obtain a minimum of 45 million reads/sample. The initial QC of the sequenced raw reads (FastQC version 0.12.1, RRID:SCR_014583) was followed by the removal of low-quality and adapter sequences (Trimmomatic version 0.39, RRID:SCR_011848). The trimmed reads were then mapped to the GRCh37.p13, assembly version GCF_000001405.14 (STAR version 2.7.9a, RRID:SCR_004463). To facilitate the visualization of mapped reads by the Integrative Genomics Viewer (IGV version 2.12.3, doi: 10.1038/nbt.1754), a genome reference index was constructed (SAMtools version 1.9, RRID:SCR_002105). The mappings were used for downstream analyses like expression quantification (featureCounts

version 2.0.1, RRID:SCR_012919). MultiQC (version 1.14.0, (RRID:SCR_014982) was utilized to generate the final quality control report.

SUPPLEMENTARY FIGURES

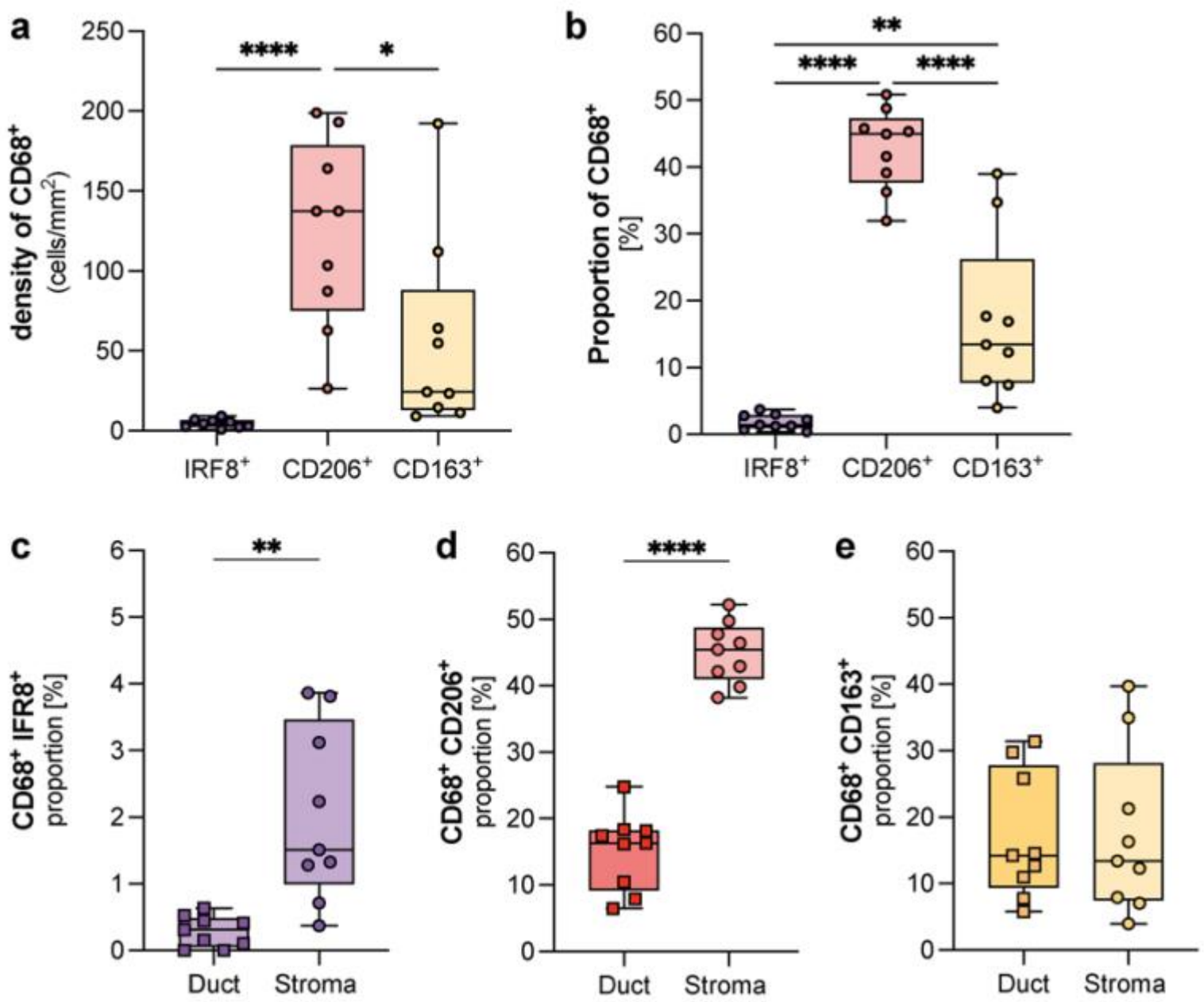

Supplementary Figures S1. Spatial analysis of CD68⁺ macrophage subsets.

(a) Box-Whisker plots depicting the median cell density (cell/mm^2) of pan macrophage marker CD68 co-expressing M1-like macrophage marker IRF8 and M2-like macrophage markers CD206 and CD163 across ductal and stromal regions (n=9). Statistical differences were identified using 2-way ANOVA; *p< 0.05, **** p< 0.0001.

(b) Box-Whisker plots depicting the frequency (%) of pan macrophage marker CD68 co-expressing M1-like macrophage marker IRF8 and M2-like macrophage markers CD206 and CD163 across ductal and stromal regions (n=9). Statistical differences were identified using 2-way ANOVA; ** p< 0.01, **** p< 0.0001.

(c-e) Box-Whisker plot depicting the frequency (%) of (c) M1-like ($CD68^+$ $IRF8^+$), M2-like (d) $CD68^+$ $CD206^+$ and (e) $CD68^+$ $CD163^+$ across ductal and stromal regions (n=9). Statistical differences were identified using one sample t test; ** p< 0.01, **** p< 0.0001.

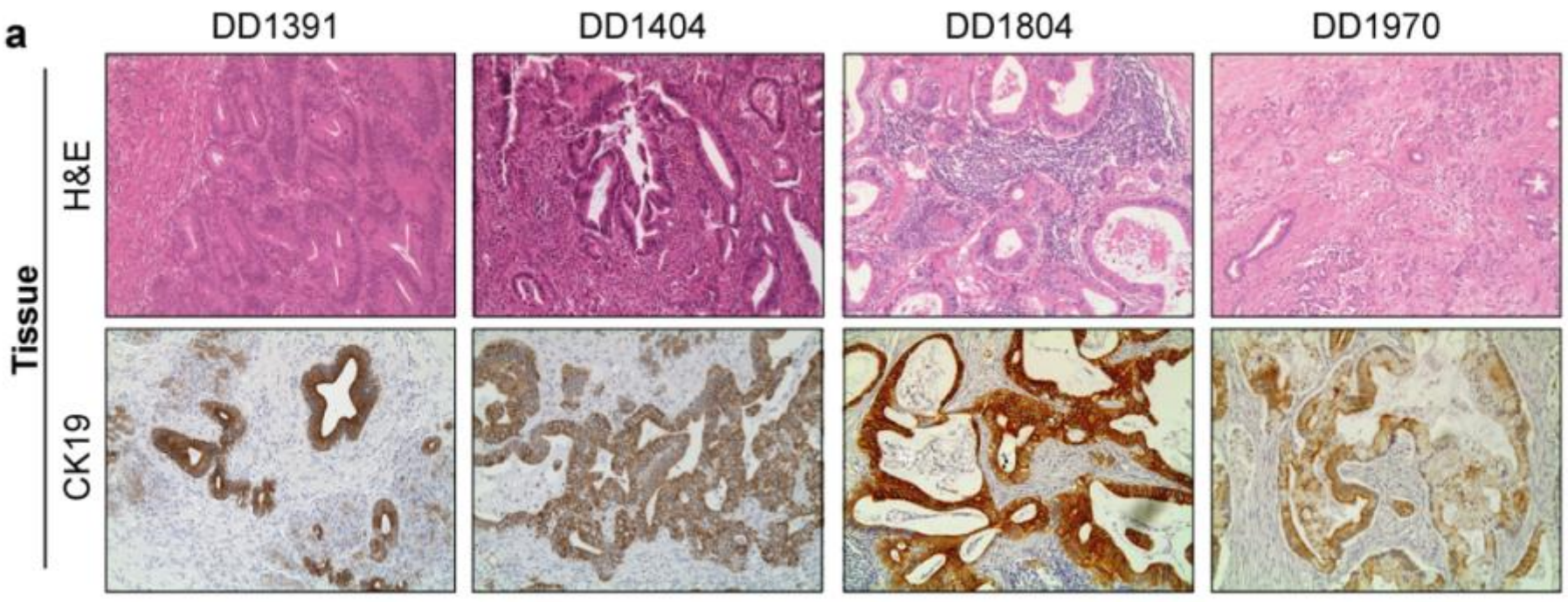

Supplementary Figures S2. Immunohistochemical analysis of PDAC tissue.

(a) Brightfield images and immunohistochemical staining (H&E, CK19) of primary PDAC tissue matching the PDOs shown in Figure 2B. Scale bar: 200µm.

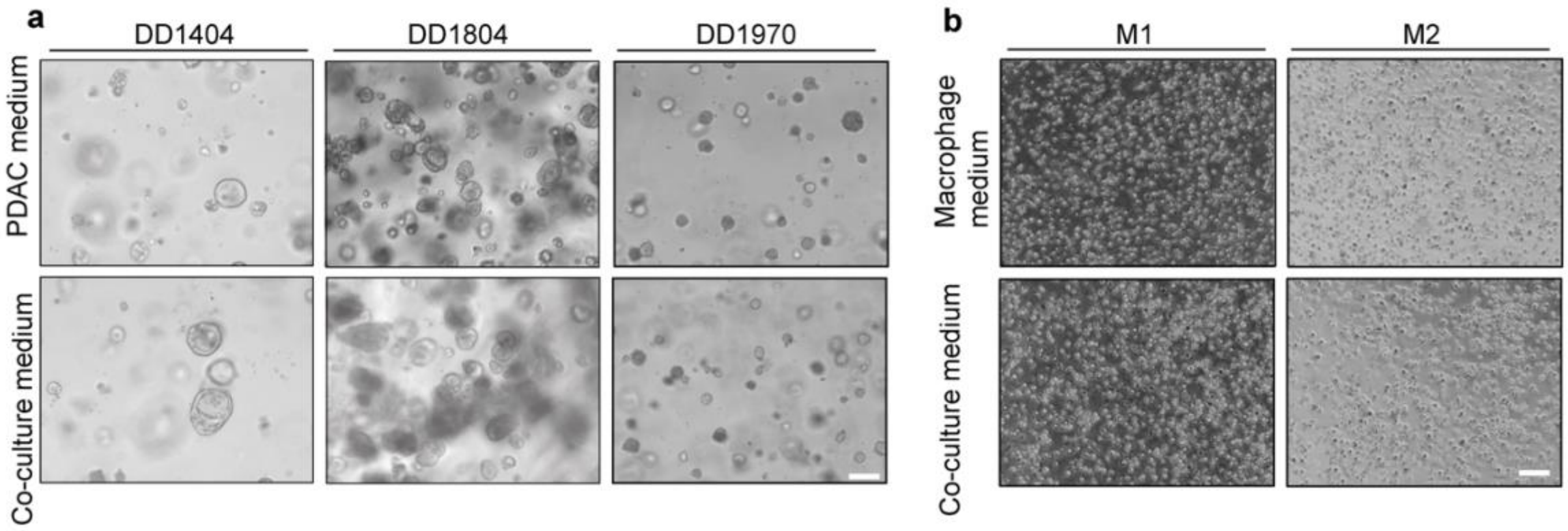

Supplementary Figures S3. Cell viability depends on culture medium.

(a) Brightfield images of Matrigel-embedded PDAC organoid lines DD1404, DD1804, and DD1970 cultured in either standard PDAC organoid medium or co-culture medium (CCM). Scale bar: 400µm.

(b) Brightfield images of M1-like and M2-like macrophages cultured in either standard macrophage medium or co-culture medium. Scale bar: 130µm.

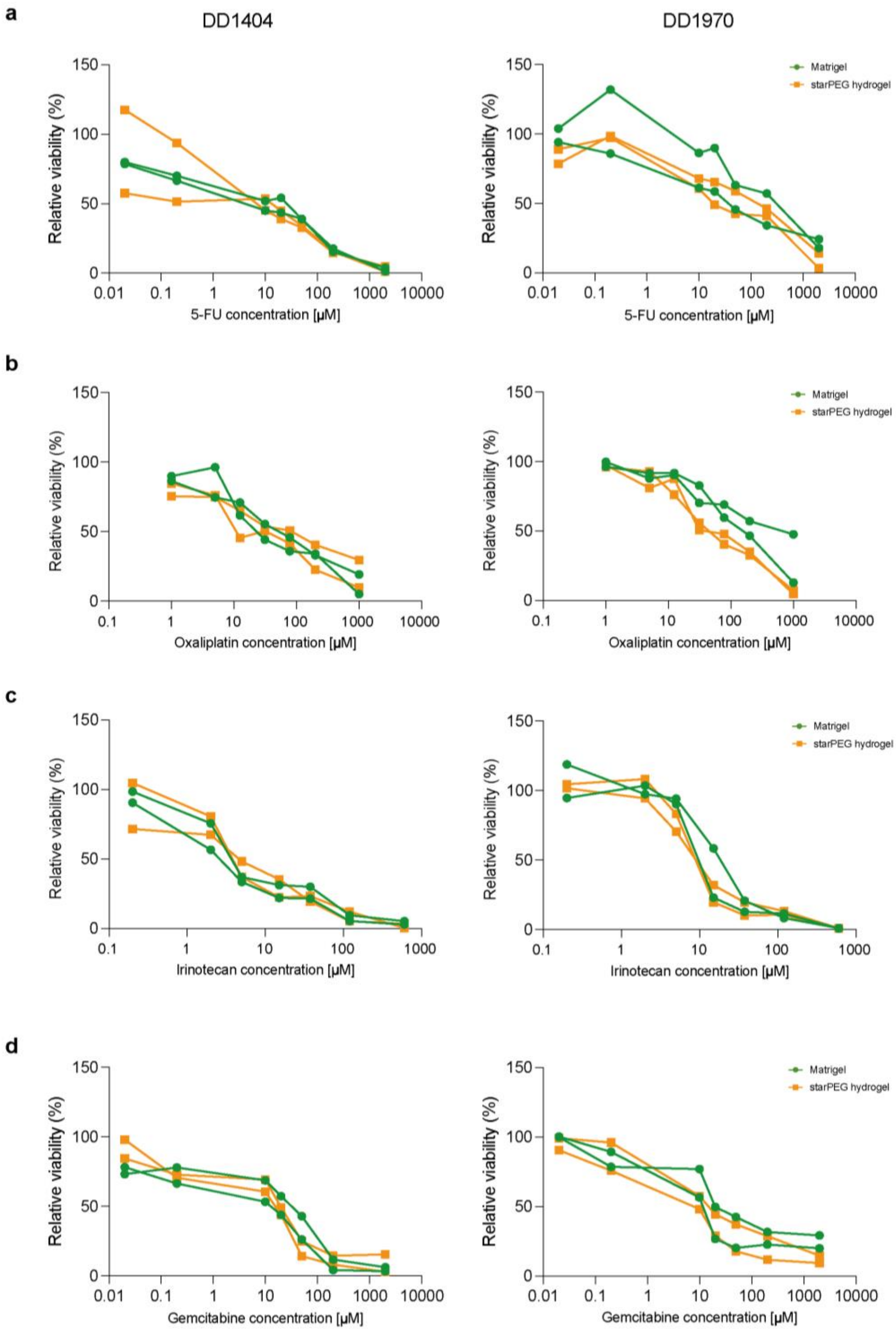

Supplementary Figures S4. PDAC PDO drug response curves to chemotherapeutic agents.

(a–d) Dose-response curves showing the relative viability of PDAC PDOs DD1404 and DD1970 cultured in either Matrigel or starPEG-heparin hydrogels following treatment with (a) 5-fluorouracil (5-FU), (b) oxaliplatin, (c) irinotecan, and (d) gemcitabine. Data are presented as replicates of n=2 independent biological replicates with n=3 technical replicates each.

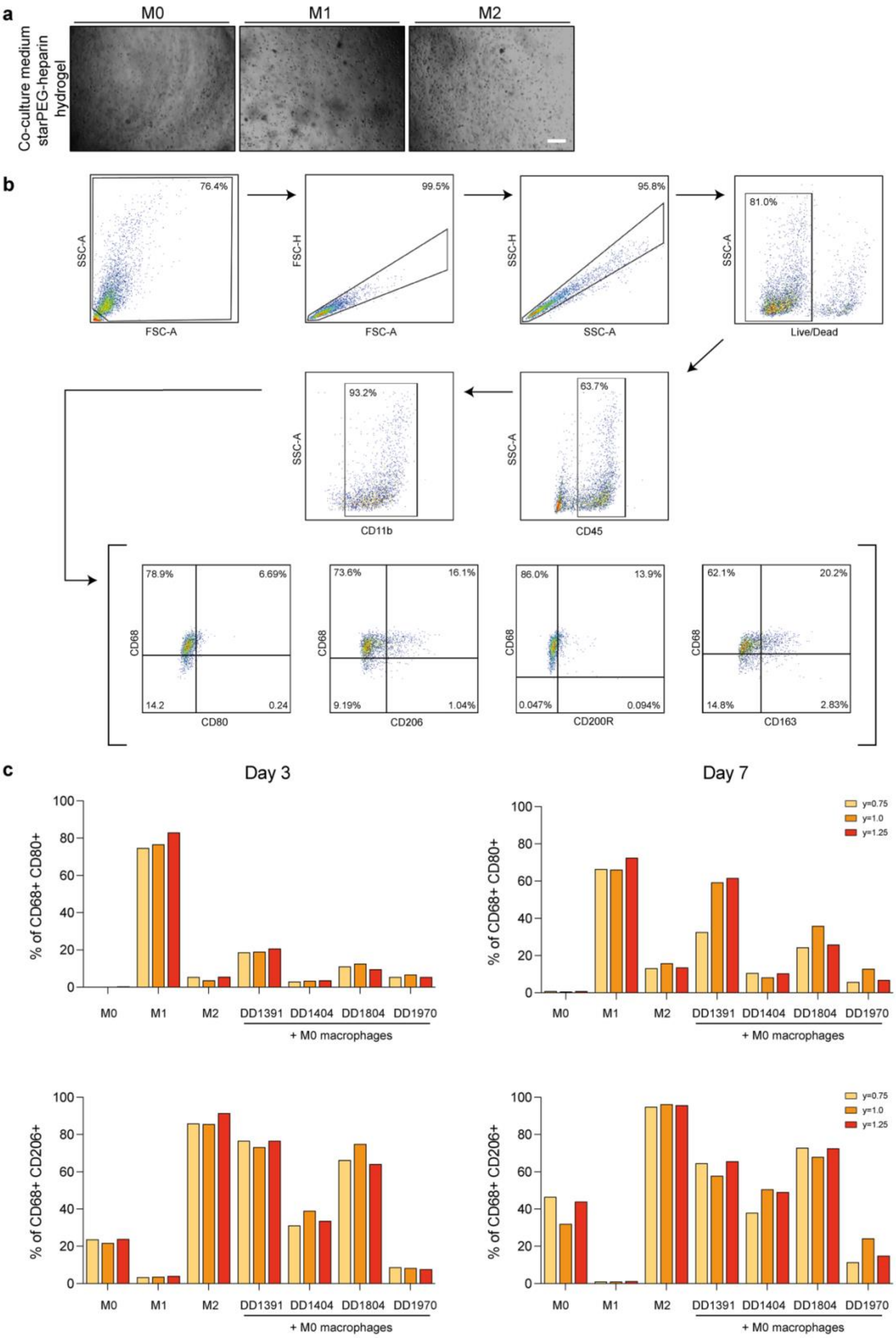

Supplementary Figures S5. Expression of known macrophage markers in macrophage monocultures and co-cultures with PDAC PDOs.

(a) Representative brightfield images of M0, M1-like and M2-like macrophages embedded in starPEG-heparin hydrogel cultured in co-culture medium. Scale bar: 130µm.

(b) Representative gating strategy for the analysis of macrophage populations. FSC-H versus FSC-A and SSC-H versus SSC-A plots were used to exclude debris and doublets. Live cells were then selected based on viability dye staining. Leukocytes were identified using the pan-leukocyte marker CD45 and myeloid marker CD11b. Under this population, macrophages were identified using the pan-macrophage marker CD68. Subsequently, the expression of macrophage surface activation markers was assessed within the CD68-positive population, including CD80, CD206, CD200R, and CD163. FMO or isotype controls for intracellular and extracellular markers were used to set appropriate gating thresholds.

(c) Flow cytometric analysis of *in vitro* differentiated M0, M1-like, and M2-like macrophages and 3D co-cultures of PDAC PDOs with M0 macrophages embedded in starPEG-heparin hydrogels exhibiting three stiffnesses: softer (γ=0.75), intermediate (γ=1.0), and stiffer (γ=1.25). Expression of the polarization-associated markers CD80 and CD206 was assessed on day3 and day7. Data are derived from a single independent experiment (n=1).

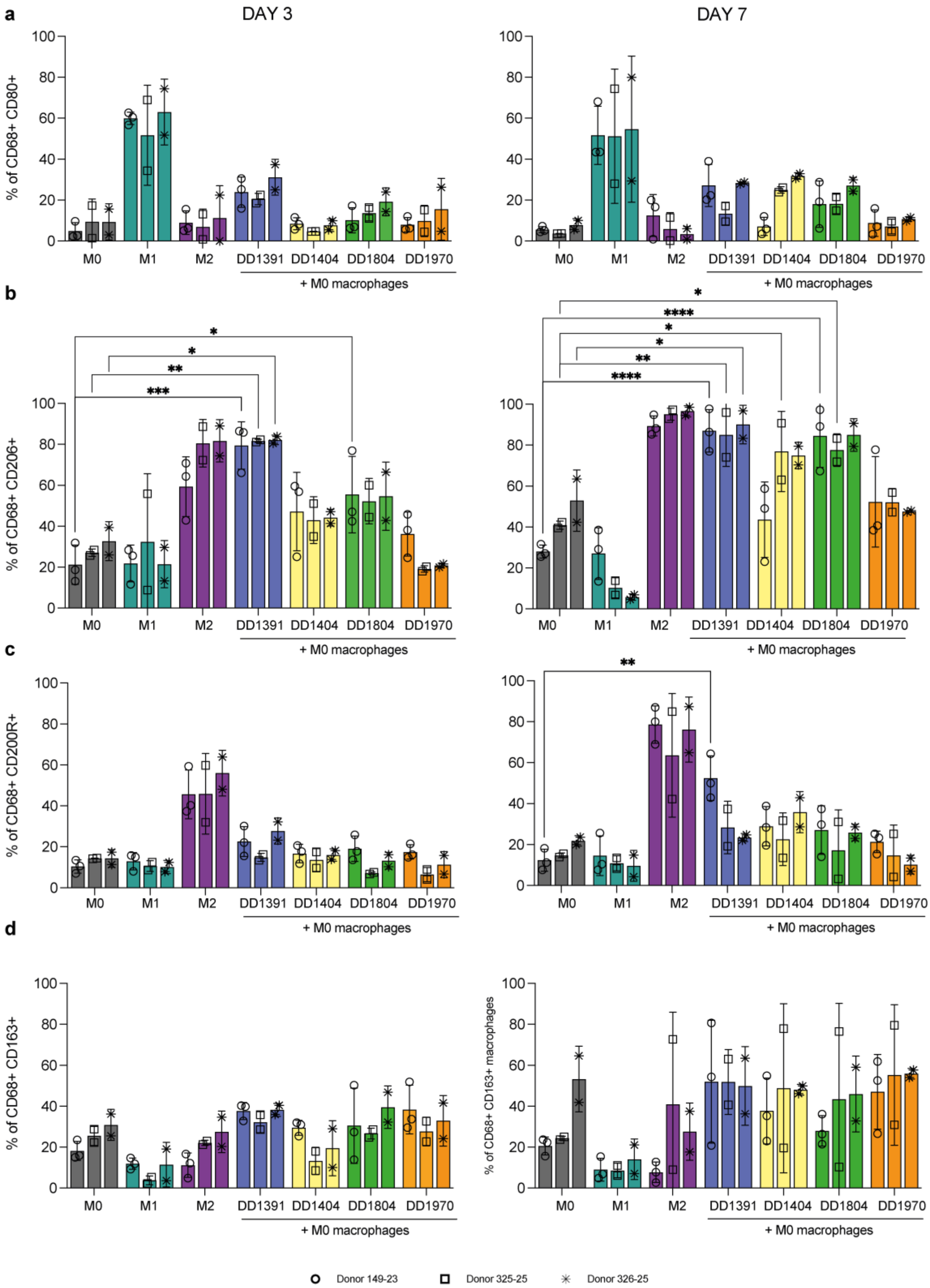

Supplementary Figures S6. Macrophage marker expression across multiple donors

(a-d) Flow cytometric analysis of *in vitro* differentiated M0, M1-like, and M2-like macrophages and 3D co-cultures of PDAC PDOs with M0 macrophages embedded in starPEG-heparin hydrogel matrix. Analysis was performed after day3 and day7 of culturing. Shown are the percentages of (a) $CD68^+CD80^+$ macrophages, (b) $CD68^+CD206^+$ macrophages, (c) $CD68^+CD163^+$ macrophages, and (d) $CD68^+CD200R^+$ macrophages. Symbols indicate macrophages derived from three independent donors (circle=donor 149-23, square=325-25, and asterisk=326-25). The mean values and SD of n=3 (Donor 149–23) and n=2 (Donors 325–25 and 326–25) independent biological replicates are displayed. ANOVA, *p<0.05, **p<0.01, ***p<0.001, ****, p<0.0001.

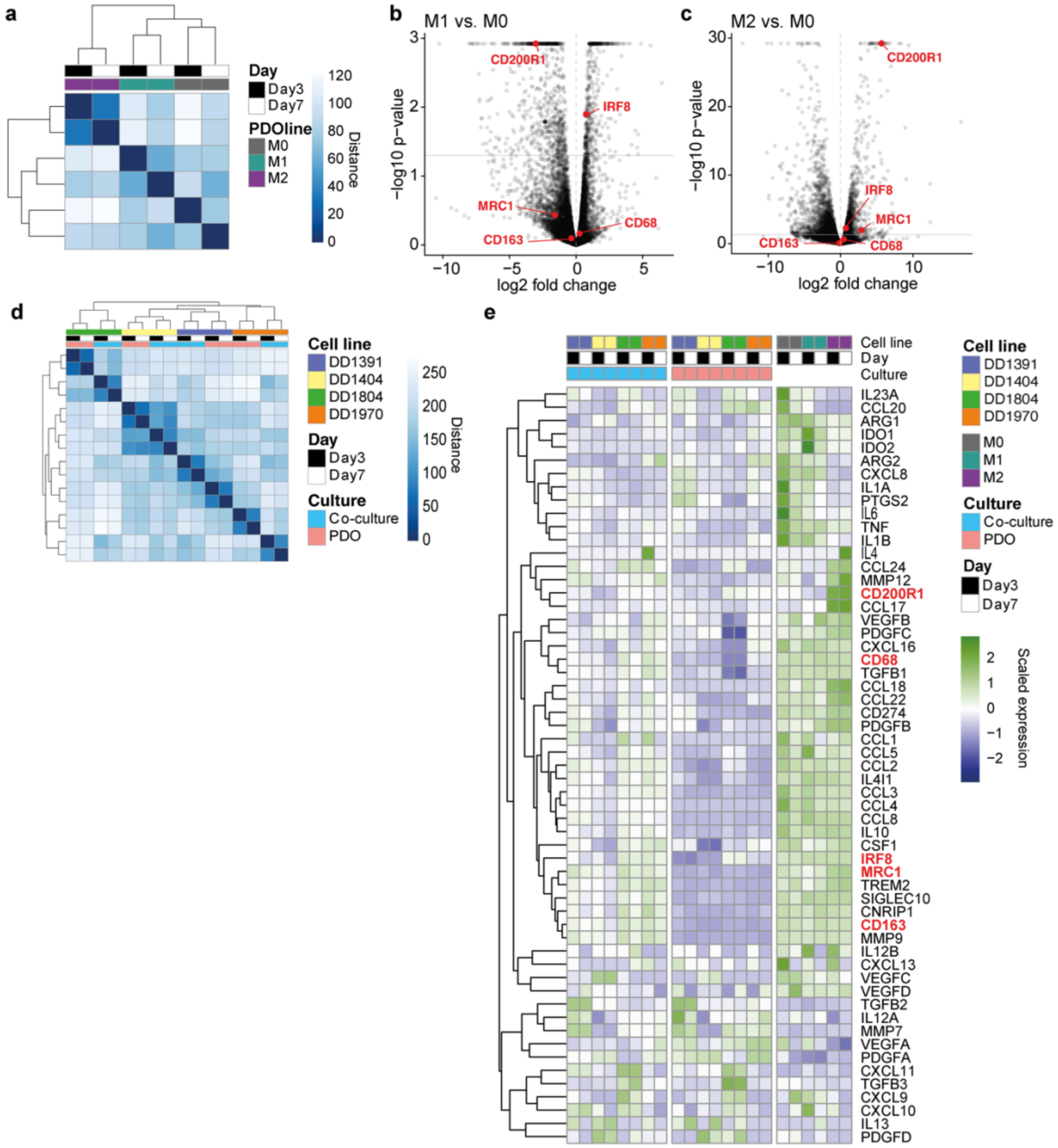

Supplementary Figures S7. Hierarchical clustering and differential analysis of macrophage monocultures and PDAC organoid-macrophage co-cultures.

(a) Hierarchical clustering of *in vitro* differentiated M0-like, M1-like, and M2-like macrophages.

(b) Differential analysis of M1-like versus M0-like macrophages. P-values represent the adjusted p-values from the differential gene expression analysis. 5 marker genes

are included that represent general macrophages (*CD68*), M1-like (*IRF8*), M2-like (*MRC1* & *CD200R1*), and tumor-associated macrophages (TAM; *CD163*). For visibility, the y-axis was capped at the highest highlighted gene with all higher values plotted at this maximum.

(c) Differential analysis of M2-like versus M0-like macrophages. P-values represent the adjusted p-values from the differential gene expression analysis. 5 marker genes are included that represent general macrophages (*CD68*), M1-like (*IRF8*), M2-like (*MRC1* & *CD200R1*), and tumor-associated macrophages (TAM; *CD163*). For visibility, the y-axis was capped at the highest highlighted gene with all higher values plotted at this maximum.

(d) Hierarchical clustering of PDOs and co-cultures.

(e) Heatmap of variance stabilized gene expression counts for a wider range of macrophage polarization-associated genes in all investigated samples. Relative gene expression counts are scaled by row. 5 marker genes are highlighted in red that represent general macrophages (*CD68*), M1-like (*IRF8*), M2-like (*MRC1* & *CD200R1*), and tumor-associated macrophages (TAM; *CD163*).

Table S1 Clinicopathological characteristics of PDAC patients.

PDAC PDO number and patient-associated parameters such as neoadjuvant treatment (NAT), tumor stage (T), lymph node stage(N), lymphatic invasion (L), vascular invasion (V), perineural invasion (P), tumor grade of differentiation (G) resection border (R).

PDO	Age	Gender	Biopsy origin	NAT	T	N	L	V	Pn	G	R	ICD-10-code	Tumor histology
DD1285	81	Female	Resection	No	1a	0	0	0	0	1	0	C 25.1	M 8500 / 3
DD1385	70	Female	Resection	No	2	1	1	0	1	3	1	C 25.0	M 8500 / 3
DD1391	61	Male	Resection	No	1c	1	0	0	1	2	0	C 25.0	M 8500 / 3
DD1404	67	Female	Resection	No	2	2	0	0	1	2	0	C 25.0	M 8500 / 3
DD1524	80	Male	Resection	No	2	1	1	1	1	2	1	C 25.0	M 8500 / 3
DD1531	73	Female	Resection	No	3	2	1	1	1	3	1	C 25.0	M 8500 / 3
DD1804	67	Male	Resection	No	2	2	1	1	1	3	0	C 25.0	M 8500 / 3
DD1970	73	Male	EUS-FNA	No	4	NA	NA	NA	NA	NA	NA	C 25.0	M 8500 / 3
DD2046	77	Female	Resection	Yes	3	1	0	2	1	X	1	C 25.0	M 8500 / 3

Table S2 Overview of mIHC and IHC antibodies.

Supplemental Table S2 Overview of antibodies

Antibody	Application	Dilution	Incubation temp, time	Fluorophor	Final Dilution	Clone	Supplier	RRID
PanCK	mIHC	Pre-diluted	36 °C, 32 min	620	1:500	2GV6	Roche	Cat# 05267145001,
IRF8	mIHC	1:300	RT, 16 min	520	1:300	EPR6855	Santa Cruz Biotechnology	Cat# sc-365042, RRID:AB_10850401
CD68	mIHC	1:300	36 °C, 32 min	540	1:300	SP57	Cell Signaling Technology, Inc.	Cat# 76437, RRID:AB_2799882
CD163	mIHC	Pre-diluted	RT, 32 min	570	1:200	236A/E7	Roche	Cat# 5973929001,
CD206	mIHC	1:50	36 °C, 32 min	690	1:50	D13C9	Cell Signaling Technology, Inc.	Cat# 91992, RRID:AB_2800175
CK19	IHC	Pre-diluted	4 °C, overnight	NA	1:100	BA16	Abcam	Cat# ab20210, RRID:AB_445429
TP53	IHC	Pre-diluted	4 °C, overnight	NA	1:200	1C12	Cell Signaling Technology, Inc.	Cat# 2524, RRID:AB_331743
Fixable Viability Stain 780	Flow Cytometry	Pre-diluted	4 °C, 15 min	APC-H7	1:1000	NA	BD Biosciences	Cat# 565388, RRID:AB_2869673
CD11b	Flow Cytometry	Pre-diluted	4 °C, 30 min	AF700	1:100	M1/70	BioLegend	Cat# 101222, RRID:AB_493705
CD45	Flow Cytometry	Pre-diluted	4 °C, 30 min	BUV395	1:100	HI30	BD Biosciences	Cat# 563791, RRID:AB_2744400
CD68	Flow Cytometry	Pre-diluted	4 °C, 30 min	PerCP Cy5.5	1:200	Y1/82A	BioLegend	Cat# 333814, RRID:AB_10683171
CD163	Flow Cytometry	Pre-diluted	4 °C, 30 min	PE	1:50	GHI/61	BioLegend	Cat# 333606, RRID:AB_1134002
CD206	Flow Cytometry	Pre-diluted	4 °C, 30 min	PE Cy7	1:50	19.2	Thermo Fisher Scientific	Cat# 25-2069-42, RRID:AB_2573426
CD80	Flow Cytometry	Pre-diluted	4 °C, 30 min	FITC	1:100		BD Biosciences	Cat# 557226, RRID:AB_396605
CD200R	Flow Cytometry	Pre-diluted	4 °C, 30 min	APC	1:33.3		BioLegend	Cat# 329308, RRID:AB_2564351

Table S3 Overview of medium components.

PDAC media components	Manufacturer	Final concentration	Volume (μL)
Advanced DMEM/F12 *	Gibco	Not applicable	12,190 µL
Wnt (100x)	**	50x	25,000 µL
Rspondin (100x)	**	10x	5,000 µL
Noggin (100x)	**	10x	5,000 µL
B27 (50x)	Invitrogen	1x	1,000 µL
Nicotinamide (1 M)	Sigma-Aldrich	10 mM	500 µL
Gastrin (100 µM)	Sigma-Aldrich	10 nM	500 µL
N-Acetyl-L-cysteine	Sigma-Aldrich	1 mM	100 µL
Primocin	Invivogen	1x	100 µL
mEGF (500 µg/mL)	Invitrogen	50 ng/mL	50 µL
hFGF-10 (1 mg/mL)	Peprotech	100 ng/mL	50 µL
A83-01 (25 mM)	Tocris Bioscience	0.5 µM	10 µL
N2 (100x)	Invitrogen	1x	500 µL

** Basal medium is supplemented with Glutamax (1:100), Pen/Strep (1:100), and HEPES 1M (1:100).*
*** These components are produced/manufactured in house.*

Macrophage medium components	Source	Final concentration	Volume (µL)
RPMI 1640 with L –Glutamine *	Gibco	Not applicable	36,000 µL
Human serum	Sigma-Aldrich	10%	4,000 µL
Human M-CSF (25 µg)	Miltenyi Biotec	25 ng/mL	10 µL

** Basal medium is supplemented with Pen/Strep (1:100).*

Co-culture medium components	Source	Final concentration	Volume (µL)
Advanced DMEM/F12 *	Gibco	Not applicable	2,800 µL
Wnt (100x)	**	25x	5,000 µL
Rspondin (100x)	**	5x	1,000 µL
Noggin (100x)	**	5x	1,000 µL
B27 (50x)	Invitrogen	0.5x	200 µL
RPMI 1640 with L –Glutamine ***	Gibco	Not applicable	9,700 µL
Human serum	Sigma-Aldrich	1.50%	4,000 µL

** Basal medium is supplemented with Glutamax (1:100), Pen/Strep (1:100), and HEPES 1M (1:100).*
*** This component is produced/manufactured in house.*
**** Basal medium is supplemented with Pen/Strep (1:100)*
